# Comprehensive network of stress-induced responses in *Zymomonas mobilis* during bioethanol production: from physiological and molecular responses to the effects of system metabolic engineering

**DOI:** 10.1186/s12934-024-02459-1

**Published:** 2024-06-18

**Authors:** Shaqayeq Asefi, Hoda Nouri, Golchehr Pourmohammadi, Hamid Moghimi

**Affiliations:** Department of Microbial Biotechnology, School of Biology, College of Science, University of Tehran, Tehran, Iran

**Keywords:** Bioethanol fermentation stress condition, Metabolic engineering, Stress response regulatory network, Synthetic biology, Systems biology, *Zymomonas mobilis*

## Abstract

Nowadays, biofuels, especially bioethanol, are becoming increasingly popular as an alternative to fossil fuels. *Zymomonas mobilis* is a desirable species for bioethanol production due to its unique characteristics, such as low biomass production and high-rate glucose metabolism. However, several factors can interfere with the fermentation process and hinder microbial activity, including lignocellulosic hydrolysate inhibitors, high temperatures, an osmotic environment, and high ethanol concentration. Overcoming these limitations is critical for effective bioethanol production. In this review, the stress response mechanisms of *Z. mobilis* are discussed in comparison to other ethanol-producing microbes. The mechanism of stress response is divided into physiological (changes in growth, metabolism, intracellular components, and cell membrane structures) and molecular (up and down-regulation of specific genes and elements of the regulatory system and their role in expression of specific proteins and control of metabolic fluxes) changes. Systemic metabolic engineering approaches, such as gene manipulation, overexpression, and silencing, are successful methods for building new metabolic pathways. Therefore, this review discusses systems metabolic engineering in conjunction with systems biology and synthetic biology as an important method for developing new strains with an effective response mechanism to fermentation stresses during bioethanol production. Overall, understanding the stress response mechanisms of *Z. mobilis* can lead to more efficient and effective bioethanol production.

**Graphical Abstract:**

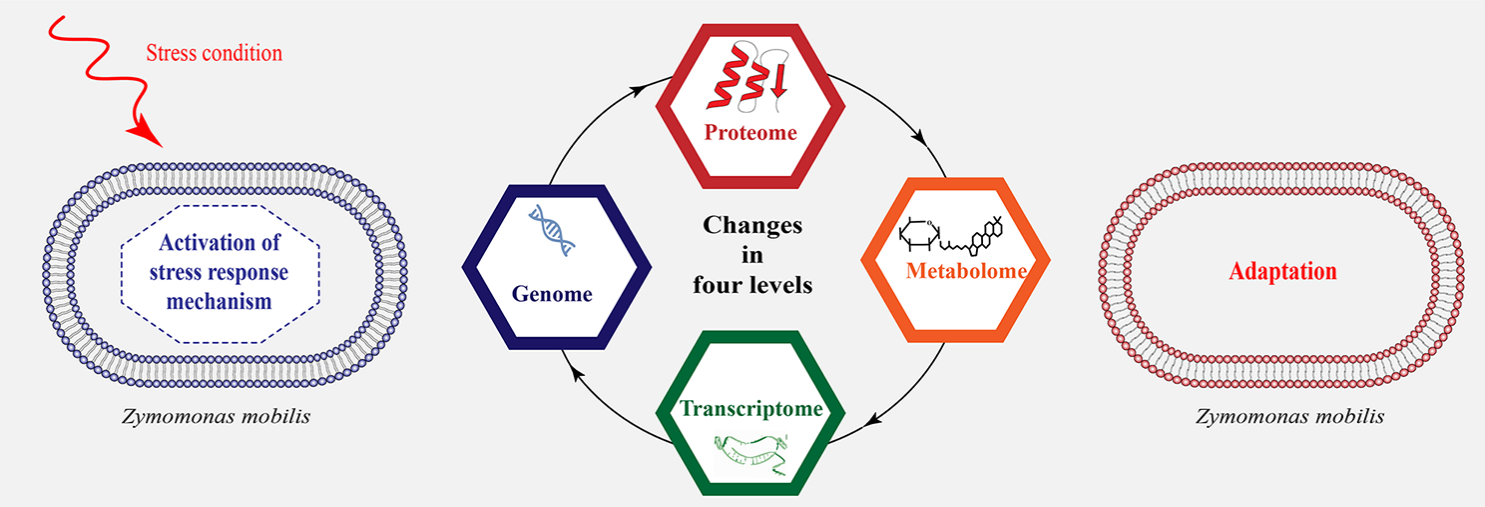

## Background

Currently, there is a potential threat of air pollution and health problems due to the emission of greenhouse gases. Therefore, the search for a preferred energy source is imperative. Among the potential energy sources, bioethanol has received great attention [1]. Bioethanol is derived from three main feedstocks, sugar, starch, and lignocellulosic biomass. In addition to indigenous strains of ethanol-producing microorganisms (*Zymomonas mobilis* and *Saccharomyces cerevisiae*) [2], such microorganisms can also include halophilic (e.g., *Candida* sp.) [3] or thermotolerant species (e.g., *Kluyveromyces marxianus*) [4] as well as genetically or metabolically engineered bacterium (e.g., *Escherichia coli* and *Scheffersomyces stipitis*) [5, 6]. Nevertheless, *Z. mobilis*, is a highly regarded bioethanol producer and some of its characteristics, including high specific ethanol yields and productivity, and more efficient ATP formation, make it a valuable strain for industrial use and research compared to *S. cerevisiae* [7].

The fermentation process exposes microorganisms to a variety of cellular and process stress conditions, many of which negatively affect cell viability and ethanol yield [8]. Most cellular-related stress conditions are due to situations related to cellular metabolism, such as the accumulation of ethanol and oxidative stress. Process-related stress factors include a variety of obstacles such as high temperatures, nutrient deficiencies, and impurities [9, 10].

**Fig. 1:**
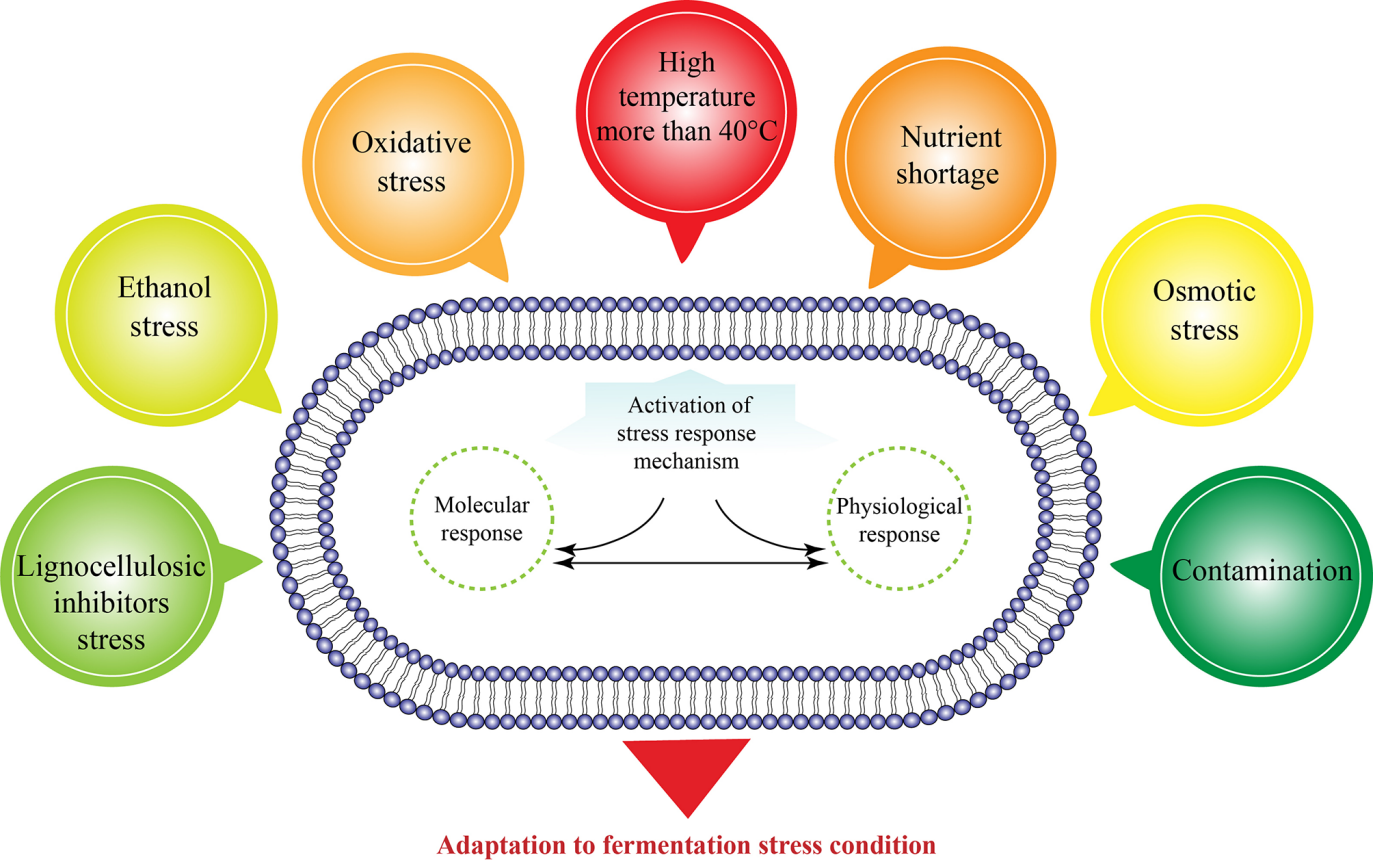
Bioethanol fermentation stress conditions and adaptation. Schematic view of process-related stress (osmotic, high temperature, lignocellulosic inhibitors, nutrient shortage, contamination with other microorganisms) and cellular-related stress (ethanol and oxidative stress) situations during bioethanol production that leads to activation of stress response mechanism in *Z. mobilis*

Stress response systems within microorganisms can interact with each other through complex global regulatory networks, allowing the cell to respond simultaneously to different types of stress [11]. According to this concept, cellular responses to these environmental fluctuations are divided into two categories: physiological responses and molecular responses. It should be noted that both types of reactions are interrelated. The physiological response is a network of cellular activities that cause physiological changes, including altering the phospholipid composition and properties of the membrane, increasing the content of specific amino acids and sugars, altering the growth rate and bioethanol production, modulating ion exchange processes, and altering the ATPase activity of the plasma membrane to conserve energy. All of these physiological responses help the cell resist and counteract stress-induced damage [12]. Molecular responses are mediated by a number of regulatory proteins and transcription factors that control gene expression, so changes in the expression of these proteins can indirectly lead to phenotypic changes and ultimately physiological changes in the cell [13]. Therefore, it is necessary to understand the cellular mechanism that contributes to overcoming these stress situations in order to improve the high level of stress-tolerant microorganisms. The general stress situations that *Z. mobilis* may be exposed to during bioethanol production are shown in Fig. 1.

Although *Z. mobilis* has inherent properties to function appropriately under a variety of stresses, there are also some engineering techniques such as adaptive laboratory evolution [14], global transcription machinery engineering (gTME) [15], and genome shuffling [16] that have been successfully used in *Z. mobilis* species adapted to furfural, acetic acid, and ethanol stresses [15–17]. With the advent of biological systems and the advances in computer models, it is now possible to target genetic and metabolic changes, resulting in desirable strains with high tolerance to stress conditions. Interestingly, systems metabolic engineering has also been able to play an important role in increasing the production of products and macromolecules [18].

This review is intended to provide an overview of the stress conditions to which *Z. mobilis* is exposed during bioethanol production and the mechanisms for overcoming these cellular barriers from physiological and cellular aspects. Accordingly, we have discussed in the following concepts: (1) the physiological responses under stress conditions, especially related to cell membrane lipid composition, growth rate and ethanol production; (2) regulatory network elements (regulatory RNAs and proteins) controlling gene expression and metabolism; (3) metabolic and genetic engineering approaches applied to obtain stress-resistant strains. Furthermore, to achieve a better understanding of *Z. mobilis* performance under industrial stress situations, we compared *Z. mobilis* with other potential ethanol-producing microorganisms (bacteria and yeast), that their stress response mechanisms were thoroughly determined and presented in previous reports.

### Effect of stress conditions on physiology and characteristics of *Z. mobilis*

#### Ethanol stress condition

Due to the increase in ethanol concentration at the end of the fermentation process, this product is one of the most common stresses in the ethanol production industry. Moreover, reactive oxygen species like superoxide and peroxide increase during the ethanol fermentation process. These compounds are toxic, and their generation is related to Fe-S containing enzymes in the respiratory chain. High concentrations of ethanol lead to ethanol stress, which increased ROS concentration and finally encountered cells with an oxidative stress condition [9]. Inhibition of cell growth, reduction in cell volume and growth rate occur at low ethanol concentrations, while at relatively high ethanol concentrations there is a decrease in cell vitality and an increase in cell death [19]. Therefore, increasing membrane fluidity, low glucose utilization, and energy production that hinder microbial growth and metabolism are examples of changes in ethanol stress conditions.

#### Effects on cell membrane structure and composition

One of the physiological changes in the action of alcohols such as ethanol is the negative effect on the cell membrane and fluidity. Therefore, it seems that strengthening the membrane is a crucial way to counteract the harmful effect of a high ethanol concentration [20].

Lipidomics analysis of the membrane composition of *Z. mobilis* revealed a high level of vaccenic acid composition in the logarithmic phase and lower levels of myristic acid, palmitic acid, and palmitoleic acid. This composition changes under ethanol stress so that the content of glyceric, palmitic, and stearic acids increases with a significant reduction in lipid/ protein content [20]. In addition, studies indicate that vaccenic acid, an unsaturated fatty acid, accounted for more than 75% of the lipid structure under normal conditions, whereas this amount decreased significantly under ethanol stress. The content of cardiolipin and phosphatidylcholine in the cell membrane also increased, while phosphatidylethanolamine and phosphatidylglycerol, the most abundant glycolipids in the cell membrane, decreased under such stress conditions [21]. For other ethanol-producing strains such as *S. cerevisiae*, under normal conditions, the primary fatty acids in the cell membrane are palmitic acid, palmitoleic acid, oleic acid, and stearic acid, whereas under stress conditions, the amount of phosphatidylserine increases [22]. Studies by Dombek et al. on *E. coli* showed high levels of vaccenic acid, low levels of palmitic acid, and a decrease in the ratio of phospholipid/protein components in the presence of 4% ethanol [23]. it can be inferred that changes in lipid composition are an evolutionary adaptation that increases survival under ethanol stress. According to these results, it appears that the unsaturated/saturated (U/S) ratio decreases during ethanol stress as a response mechanism to control and reduce membrane fluidity. In a study by Huffer et al. the content of unsaturated and saturated fatty acids was investigated in the presence of different ethanol concentrations in *E. coli* K12, *Z. mobilis*, *K. marxianus* and *S. cerevisiae*. The results show that with increasing ethanol content, the ratio of U/S decreases. In *Z. mobilis* and *E. coli* K12, this decrease was about 60% and 40%, respectively [24].

Hopanoids in bacteria such as *Z. mobilis* and ergosterols in yeast species such as *S. cerevisiae* and *K. marxianus* are important sterol-like compounds that control membrane function. They appear to exert a strong influence on membrane properties by increasing van der Waals forces between lipid molecules and decreasing the penetration of small molecules. Therefore, this property is advantageous for the living cells at high ethanol concentration. For example, in *S. cerevisiae*, genes such as *erg24*, *erg3*, and *erg2* (responsible for ergosterol biosynthesis) are up-regulated under stress conditions with ethanol concentration higher than 10% [25]. On the other hand, in *K. marxianus*, the expression of genes such as *erg3* (C-5 sterol desaturase), *erg11* (lanosterol 14-α-demethylase), and *erg25* (C-4 methyl sterol oxidase) decreased in the presence of 6% ethanol [26]. In addition, hopanoids in *Z. mobilis* account for about 30% of the bacterial dry weight [24, 27]. The abundance of hopanoids and their polar head groups, the upregulation of genes, including *hnpA* and *hnpB*, responsible for hopanoid biosynthesis in *Z. mobilis* at 5% ethanol concentration, and the high content of tetrahydroxybacteriohopane (THBH) and hopanols at high ethanol concentration appear to maintain membrane stability and play a key role in determining ethanol tolerance [27]. Despite some studies suggesting an increase in hopanoid biosynthesis-related genes, the total concentration of terpenoids decreased upon ethanol stress in *Z. mobilis* [21]. Thus, there are still controversial hypotheses about the role of these types of terpenoids in the response to ethanol [20, 21]. Consequently, a high content of saturated and unbranched fatty acids in combination with complex structures such as glycolipids and steroids increases the ability of membranes to resist the negative effects of ethanol. Table 1 summarizes the studies reported on *Z. mobilis*, *S. cerevisiae*, *E. coli*, and *K. marxianus* during ethanol stress and compares the changes of lipid composition in these microorganisms.

**Table 1:** Physiological and molecular stress response in *Z. mobilis* compared to other ethanologenic species

Stress Condition	Microorganisms	Physiological response	Regulatory response	References
Ethanol stress	*Z. mobilis*	- Changes lipid composition, - decrease lipid/protein ratio - decrease vaccenic acid content - increase in phosphatidyl choline, stearic acid, palmitic acid - decrease unsaturated/saturated lipid ratio - increase hopanoids content - accumulation of sorbitol - up-regulation of tryptophan operon - decrease cell growth - decrease bioethanol production, up- regulation of *gfo* gene	ZliE, ZliS, MarR family, XRE family, sigma-E, sigma 70, sigma 54, sigma 28, HxIR family, TetR family, LysR family, RpiR family, PspA, PspB, PspC, Zms2, Zms18, Zms6	[20, 21, 30, 34, 73, 79, 88]
	*E. coli*	- change lipid composition - decrease lipid /protein content - decrease unsaturated/ saturated lipid change cell shape - decrease growth rate, decrease bioethanol production	groS, groL, grpE, and dnaK	[24, 29]
	*S. cerevisiae*	- changes lipid composition - increase phosphatidyl serine content in cell membrane - increase ergosterol content - accumulation of trehalose -change cell volume -increase tryptophan and proline in cytoplasm -upregulation of *tps* gene	Hsp40 (Ydj1), Hsp70 (Ssa1), SSE1, SSE2, Bip, KAR2, Hsp90 (Hsp82, Hsc82), Hsp104, SHFs, SSA2, UBR1, UBCH5, CTT1, Hsp12, Hsp30, Hsp26	[19, 22, 24, 25, 33, 87]
	*K. marxianus*	- decrease unsaturated/saturated ratio -decrease ergosterol content -inhibit cell growth -decrease bioethanol production -down regulation of *erg* genes - down-regulation of genes relate to fatty acids biosynthesis	Hsp78, Hsp26, Hsp60, Hsp12, Gtt1, Pre1, Pre7, Rpn6, Rpn7, Htx1, GCN4	[24, 26, 83, 86]
Lignocellulosic hydrolysate inhibitor stress	*Z. mobilis*	- decrease growth rate - decrease bioethanol production -increase expression of genes relate to PPP and TCA like Zwf - decrease membrane fluidity and integrity - decrease protein synthesis - activation of DNA repair system - increase expression of reductase enzyme - up-regulation of ABC and RND transporters -decrease in major facilitator superfamily (MFS)	Hfq, LysR, LytR, GntR, TetR, LacI, sigma-70, sigma-28, MerR family (plorR), Fis family, PspA, PspC	[36, 40, 41, 43, 93]
	*S. cerevisiae*	- decrease ribosomal synthesis -increase ABC transporters (Pdr5, Snq2) -increase expression of proteins relate to PPP (especially Zwf) and TCA - increase cytosolic stress granules and P-bodies -increase ATP protein synthesis -increase cytochromes protein -increase expression of Adh7 and Bdh2 -increase expression of Ald6	Yap1	[40, 44, 45, 52, 56, 61]
	*Scheffersomyces stipitis*	- decrease growth rate - decrease bioethanol production -inhibition of glycolysis pathway -increase PPP and TCA pathways and their enzymes activity (Cit1, Idp2) -decrease expression of tdh gene (glyceraldehyde-3-phosphate dehydrogenase) - increase expression of Ino1 (inositol synthetase) - increase expression of *YBO9*, *FAA22*, *FAA24* (relate to long-chain fatty acid biosynthesis)	Haa1, proteasome assembly chaperone (Tma17), Mbf1, Hsp70 (Ssa2.2)	[40, 45]
High temperature	*Z. mobilis*	-decrease bioethanol production -decrease growth rate -elongated shape -accumulation of sorbitol -increase activity of ADH and PDC enzyme -activate cell division proteins -changes lipid composition -up-regulation of DNA repair system	Zn- dependant peptidase containing M16 subunit, DegP- protease (chaperone/ serine protease), Transcriptional regulator (WrbA), mHsp70	[44, 52, 61, 62]
	*S. cerevisiae*	- decrease ethanol production - decrease growth rate - accumulation of trehalose - accumulation of glycogen - up-regulation of genes encoding glyceraldehyde-3-phosphate dehydrogenase, hexokinase, alcohol dehydrogenase	Hsp26, SSA4, Hsp82 (Hsp90), Hsp104, Rsp5 (ubiquitin ligase), Hsp30, Hsp60, Hsp42, Cpr, Sit1, Zpr	[56, 63]
	*P. kudriavzevii*	- decrease ethanol production - decrease growth rate - accumulation of trehalose - metabolism of glycogen -up-regulation of *adh* and *tdh2* gene	Ssq1, Hsp90	[60]
High glucose concertation and osmotic stress	*Z. mobilis*	- decrease bioethanol production - decrease cell growth - upregulation in membrane channels and proteins such as Ton transporter system, ABC transporters, Type I secretion system, chloride channel protein, and Mg^2+^ transmembrane protein - accumulation of sorbitol in cytoplasm - highly expression of *gfo* gene - increased activity of GFOR enzyme in the presence of sucrose or fructose and glucose as C source - reduction in expression of *pdc* and *adh* - inhibit cell viability - decrease protein synthesis	Sigma32, catalase, sigma54 specific transcriptional activator psp54, DSBA oxidoreductase, stress responsive alpha- beta barrel domain proteins, Hsp20, DnaJ, PspA, PspB, LysR family, AsnC family, Hx1R family,	[65, 69]
	*S. cerevisiae*	-activation of CWI signaling pathway - accumulation of glycerol - increased expression of *gpd* gene - increased GPD (Glycerol-3-phophate dehydrogenase) enzyme activity - increased expression of aquaporin membrane proteins - increased in Fps1p membrane protein	Hsp ptoteins (regulated), SSA4 (not regulated)	[67, 71, 95]
	*K. marximus*	- accumulation of glycerol - inhibit catalytic activity - lowering expression of hexokinase and glucokinase enzyme - up-regulation of GPD enzyme - increased expression of low-affinity hexose transporters such as Rag1p, Kht1p	Mig1, Hsp26, Hsp42, Hsp31, Hsp12	[67, 68]

#### Effects on growth rate and metabolism

In addition to lipid composition, ethanol stress can also affect bacterial growth rate and glucose consumption. Under ethanol stress conditions, the growth rate of *Z. mobilis* decreases, significantly. A study by He et al.on *Z.mobilis* showed that the bacterium could not grow in the presence of 5% ethanol concentration [21]. When comparing ethanol-treated and untreated cells, it was found that ethanol-treated cells reached their maximum cell density in longer periods than untreated cells [21]. Comparison of growth inhibition in four species, including *E. coli* K12, *Z. mobilis*, *S. cerevisiae*, and *K. marxianus*, showed that *Z. mobilis* could tolerate a higher ethanol concentration than the other species [24].

As shown in *Z. mobilis*, most metabolic pathways are slowed in response to stressful conditions [20]. In addition, changes in gene regulation were observed under stress conditions. Most up-regulated genes were related to energy production and stress response proteins, whereas most down-regulated genes were related to translation and ribosomal structure [20, 21]. It can be concluded that the decrease in glucose consumption and bioethanol production might be related to the lower expression of enzymes responsible for glycolytic processes. In addition, the destruction of the membrane leads to a loss of water, cofactors and intermediates necessary for the activity of the enzyme [24]. Growth inhibition was also observed in other strains. In *S. cerevisiae* and *E. coli*, ethanol stress conditions affected cell division, growth, and viability in such a way that high ethanol titers resulted in cell death in *S. cerevisiae* and rounded and swollen cells in *E. coli* [28, 29]. It seems that in this case the changes in the structures somehow correlate with changes in the structure of the lipid bilayer. According to this concept, variations in lipid membrane content significantly affect selective permeability and alter cellular efflux, leading to changes in cell turgor pressure and cell shape.

#### Effects on cellular enzymes and metabolites

In ethanol-producing strains, the accumulation of sugars and amino acids also appears to be a physiological response to ethanol stress. Studies on the role of sugars in the response to ethanol stress in *S. cerevisiae* show that genes encoding TPS1 and TPS2, which are responsible for trehalose biosynthesis, are upregulated under in such conditions [25]. Glucose-fructose oxidoreductase (GFOR) expression, which is responsible for sorbitol production, is also increased in *Z. mobilis* in response to ethanol stress [30]. Accumulation of sorbitol and trehalose in the cytoplasm with the participation of Hsp proteins seems to stabilize proteins and prevent their denaturation [25, 30, 31]. In addition, amino acids can protect cells from damage caused by freezing, desiccation, and oxidative stress during the fermentation process [32]. Proline and tryptophan are important amino acids that increase cell tolerance to ethanol stress. According to some reports, the role of proline (*PRO1* gene) in ethanol tolerance has been observed in *S. cerevisiae* [33]. While the amino acid proline is necessary for ethanol tolerance in yeast, tryptophan appears to play an important role in both *S. cerevisiae* and *Z. mobilis* [19, 29]. In *Z. mobilis*, metabolomics analysis revealed that the expression of the tryptophan operon, which consists of two genes encoding this amino acid, is upregulated 2-fold [20]. The increased expression of tryptophan biosynthesis was also observed in *S. cerevisiae* as a result of high expression of TAT2, TRP2, and TRP5 genes [33]. Therefore, it should be noted that the accumulation of this compound in the cytoplasm can strengthen membrane stability and prevent protein aggregation in the cytoplasm during refolding, which is also toxic to the cell [29, 34].

(Table 1)

### Lignocellulosic hydrolysate inhibitors stress condition

Lignocellulosic biomass is one of the possible feedstocks for bioethanol production. Inhibitors formed during the pretreatment of lignocellulose are classified into five groups: Furanaldehydes, organic acids, aromatic compounds, alcohols and inorganic compounds [35]. The accumulation of these compounds in the growth culture produces a stress state for the microorganisms that impedes their activity by reducing growth rate, decreasing bioethanol production, inhibiting metabolic enzymes, and altering cell components and transcription levels [36, 37]. In addition, inhibitors, such as furfural, can induce oxidative stress by causing the accumulation of reactive oxygen species inside the cell. Consequently, this accumulation could damage various cellular components, including DNA, lipids, and proteins [38].

Cellular responses of *Z mobilis* ZM4 to representative biomass-derived inhibitors such as formic acid, acetic acid, furfural, 5-hydroxymethylfurfural, and phenol were quantified using proteomics and metabolomics methods. Changes in protein expression were observed in DNA replication, DNA recombination, DNA repair, DNA transcription, RNA translation, amino acid biosynthesis, central carbon metabolism, cell wall/membrane biogenesis, and energy production and metabolism [39].

#### Effects on DNA

The negative effects of furfural on DNA are consistent with the accumulation of ROS, including hydrogen peroxide, superoxide anions, and hydroxyl radicals, which lead to DNA damage and the destruction of proteins and lipids [38]. In *Z. mobilis*, DNA destruction is restored by upregulation of genes related to the DNA repair system and recombination, such as *dnaA*, *uvrA*, *uvrB*, *recJ*, *recF* [36].

#### Effects on lipid membrane composition

Another effect of lignocellulosic compounds on strains is the inhibition of genes related to lipid metabolism. In *Z. mobilis*, in the presence of high furfural concentration, the expression of some genes related to membrane composition decreased, including *oprM* (lipoprotein biosynthesis), *kpsC* (polysaccharide capsule biosynthesis), and flagellar proteins [36]. Other membrane compounds such as hopanoids were significantly reduced in the presence of furfural. These results suggest that furfural has a negative effect on membrane stability and that membrane integrity fluctuates under such stress [36]. One reaction mechanism to improve membrane stabilization is to increase the content of long-chain fatty acids in the membrane. This phenomenon was observed in *S. stipitis* in the presence of three lignocellulosic inhibitors (HMF, acetic acid, and vanillin). In this strain, up-regulation of genes such as *INO1* (inositol synthetase), *FAA22* (long-chain-fatty-acid CoA ligase), *FAA24* (long-chain-fatty-acid CoA ligase 2), and *YBO9* (very-long-chain 3-oxoacyl-CoA reductase), responsible for long-chain fatty acid production, enhances the ability of the microorganism to compete with these stresses [40].

#### Effects of membrane transporters

In addition to membrane proteins, there were some transporters whose expression changed markedly under stress with phenolic aldehydes in *Z. mobilis* [41]. For example, the adenine triphosphate binding cassette (ABC) and resistance-nodulation-cell division (RND) were up-regulated for about 3 and 7-fold in *Z. mobilis*, which seems to have a positive effect in exporting inhibitors out of the cell [41]. The induction of ABC transporter genes such as *snq2* and *pdr5* in *S. cerevisiae* was also observed under vanillin stress, which seems to have a positive effect on the excretion of vanillin from the cell [42].

#### Effects on proteins

Under stress conditions with lignocellulosichydrolysates (LCH), inhibition of protein synthesis was observed in most ethanologenic strains. Inhibition of protein synthesis in *Z. mobilis* and *S. cerevisiae* was studied under furfural and vanillin stress conditions [36, 42]. In *S. cerevisiae*, inhibition of protein synthesis led to the formation of stress granules and p-bodies in the cytoplasm; however, such phenomenon was not observed in *Z. mobilis* [43]. On the other hand, most of the down-regulated genes in these two microorganisms were associated with ribosomal synthesis, such as *rbfA*, *rbsR*, *rplI*, and *rpsF* in *Z. mobilis* [36] and Rpa 12, Rpa 190, and Rpc11 in *S. cerevisiae* [42, 44]. In addition, genes related to amino acid metabolism and tRNA synthesis were downregulated in the presence of lignocellulose inhibitors. These results suggest that inhibition of protein synthesis provides these microbial species with the potential energy they need to survive under such stressful conditions [44].

Although protein synthesis was inhibited under lignocellulosic inhibitor stress, the expression of some other protein increased in such situations. Most of these genes are related to carbon metabolic pathways, including the ED pathway and the tricarboxylic acid (TCA) cycle. Proteins, including Zwf (glucose-6-phosphate dehydrogenase), Adh (alcohol dehydrogenase), Ald (aldehyde dehydrogenase), Cit1 (citrate synthase), Ihd2 (isocitrate dehydrogenase), Sdh2 (succinate dehydrogenase) that play a vital role in the metabolism of carbohydrates were up-regulated [36, 43, 45]. NADH and NADPH will be produced during these processes, which seems to have a significant role in stress tolerance by controlling the redox balance.

On the other hand, NADPH produced through these mechanisms is necessary for protecting cells against ROS and oxidative stress induced by hydrolysate inhibitors. NADPH is utilized by many stress protection enzymes, including thioredoxin peroxidase and glutathione oxidoreductase [38]. Glutathione and glutathione oxidoreductase enzymes are essential for oxidative stress responses. Consequently, sulfate assimilation and cysteine biosynthesis will increase in response to hydrolysate inhibitors’ stress conditions [46].

In a study by Skerker et al., they determined that the increased demand for sulfite and cysteine is due to the heightened needs of glutathione, formed from glutamate and cysteine, during *Z. mobilis* growth in stressful conditions. They proposed that the *cysC* and *cysHIJ* genes are crucial for *Z. mobilis*’s tolerance to hydrolysates [47]. Interestingly, genes including *ZMO0426* (FeS assembly protein SufBD), *ZMO0427* (SufS subfamily cysteine desulfurase), and *ZMO0005* (encoding sulfate adenylyltransferase subunit, CysD) were up-regulated which seems to contribute to protecting *Z. mobilis* 8b cells from oxidative stress caused by furfural [48].

In addition, sulfate assimilation as a response to furfural stress has been reported in *E. coli* and *S. cerevisiae* as well. Studies on *E. coli* revealed that *cys* genes were up-regulated in response to furfural detoxification, and cysteine supplementation could alleviate furfural toxicity [49]. Moreover, cysteine supplementation resulted in furfural, acetate, and ethanol tolerance in *Z. mobilis*. This indicates that the cysteine pool is necessary in microorganisms, and GSH (a cysteine-containing tripeptide) is the required antioxidant in response to oxidative stresses induced by hydrolysate inhibitors [50].

Along with metabolic pathways leading to NADH and NADPH production, some reductases, including oxidoreductase and NADH: flavin oxidase, play a crucial role in stress factors. For example, in *Z. mobilis*, under phenolic aldehydes such as vanillin, 4-hydroxybenzaldehyde, and syringaldehyde reductase enzymes were up-regulated, resulting in a reduction of these compounds into 4-hydroxy benzyl alcohol, vanillyl alcohol, and syringyl alcohol, respectively [51].

Accordingly, it can be concluded that detoxification of vanillin, furfural, and HMF is a kind of NADH-NADPH-dependent process that can alleviate inhibitors’ toxicity in the cell [43, 45]. It seems that up-regulation in enzymes and proteins that role in metabolic pathways is among efficient physiological responses, which is also common in other ethanologenic species such as *S. cerevisiae* and *S. stipitis* (Table 1).

Therefore, during ethanol fermentation, microorganisms activate DNA repair systems, produce cofactors such as NADH and NADPH, and activate genes that produce reductase enzymes to convert lignocellulosic inhibitors into less toxic compounds and detoxify cells.

### High temperature stress condition

Ethanol fermentation is known to be an exothermic process, so the microorganisms have to cope with this high temperature [52]. Moreover, it is worth they state that high temperature fermentation allows simultaneous saccharification and fermentation (SSF). Interestingly, this process reduces the risk of contamination with other microorganisms [53]. Most ethanol-producing microorganisms, specifically *Z. mobilis* and *S. cerevisiae*, are among mesophilic species with the ability to grow in temperatures between 30–35℃ [52, 54]. Therefore, the use of microorganisms capable of reacting and growing under such stress conditions is of great importance.

Heat shock stress can adversely affect ethanologenic strains by destroying cellular macromolecules and impairing cellular metabolism [55]. In *Z. mobilis*, the increased temperature may hinder cell growth and impact cell viability, so several responses are required to adapt to this condition [52].

#### Effects on cell growth and ethanol fermentation

The first effect of increased temperature is the growth rate and ethanol production, observed in *Z. mobilis*, *S. cerevisiae*, and *P. kudriavzevii*, which is also summarized in Table 1. The results of a study by Samappito et al. showed that the growth rate of the thermally adapted strain at 37℃ and 40℃ was about 1.1 and 62.5 times higher than that of the wild type. In addition, ethanol production at 39℃ and 41℃ in thermoadapted strains was 1.8 and 38.6 times higher than wild type, respectively [44]. The thermotolerant strains of *S. cerevisiae* were able to grow at temperatures above 40℃ (normally they grow at 25–30℃) and achieved a growth rate of 43 and an ethanol yield of 9.2% [56]. From these results, it is clear that strains with the ability to respond and tolerate such high-temperature stress conditions are of great importance.

#### Effects on cell morphology and membrane composition

The physiological stress responses of *Z. mobilis* to heat shock conditions are complicated and involve it includes the expression of several genes related to membrane stabilization, transport, and cell division. In addition, high temperatures can affect cell morphology. In *Z. mobilis*, for example, elongated cells were observed at high temperatures in the wild types. This appears to be a consequence of high temperature negatively affecting bacterial DNA and inhibiting cell division. In this case, the expression of genes related to cell division, including MinD and MinC, is inactive and activates the correct placement of division sites [52]. In high temperature, *Z. mobilis* increase the expression of genes related to membrane structures and proteins as a response. For instance, overexpression of TolB and TolQ proteins seems to impact the membrane’s stability, positively [52]. In gram-negative bacteria, a *tol-pal* gene constitutes two operons of *tolQRA* and *tolBpal*. Proteins encoded by this operon are related to LPS and porins of the outer membrane, which affect membrane stability by regulating the translocation and assembly of membrane components [52, 57].

In addition, because ethanol fermentation is an exothermic process, high temperatures may expose microorganisms to oxidative stress due to the accumulation of endogenous reactive oxygen species. The accumulation of ROS in the cell can have detrimental effects, including the disruption of membrane lipids, proteins, and DNA [58]. In *Z. mobilis*, *ZmcytC* (cytochrome c peroxidase), *ZMO1573* (iron-dependent peroxidase), *Zmcat* (catalase), *Zmsod* (superoxide dismutase), and *ZmahpC* (alkyl hydroperoxide reductase) are among the important genes involved in the response to oxidative stress in *Z. mobilis* during high-temperature stress conditions. Studies have shown that *ZmCytC* catalyzes peroxidase reactions with reduced ubiquinol-1 or NADH as an electron donor in membrane fractions, accepting electrons from the bc_1_ complex via cytochrome c_552_ to convert H_2_O_2_ to water in the periplasm [59]. Therefore, ZmCytC is critical for *Z. mobilis* survival at high temperatures.

#### Effects on nucleic acid structure and DNA repair system

High temperatures can affect intracellular structures, including DNA, tRNA, and rRNA, because an increase in temperature can alter their three-dimensional structure by disrupting hydrogen bonds. In this case, the expression of genes that play a crucial role in DNA-RNA restoration, including RadA, RadC, XseA, and SpoU is critical for coping with such stress conditions [60]. The activity of these proteins reduces DNA damage and activates repair systems. In addition, as discussed earlier in this section, elongation of *Z. mobilis* cells occurs under stress conditions at high temperatures [52], in this case, the expression of genes related to cell partitioning and division, including MinD and MinC inactive potential sites and activate correct placement of division sites [44]. On the other hand, the accumulation of sugars and carbohydrates confers thermotolerance to cells in some cases. For example, the accumulation of sorbitol in *Z. mobilis* or trehalose in *S. cerevisiae* and *P. kudriavzevii* leads to the protection of proteins from denaturation, destruction, and aggregation [30, 44, 61]. The main physiological changes under high-temperature stress conditions in *Z. mobilis*, *S. cerevisiae*, and *P. kudriavzevii* are listed and compared in Table 1.

#### Effects on metabolic pathways and intracellular metabolites

High temperature fermentation also has a negative effect on enzyme activity. This negative effect is due to inhibition of genes encoding important enzymes, including PDC and ADH. A comparison between thermotolerant and wild-type strains of *Z. mobilis* showed that these genes were strongly expressed in the thermoadapted strains [62]. In such conditions, it seems that accumulation of sorbitol [30] and high expression of proteins controlling translation such as HrpB (an ATP-dependent helicase) [52] positively influence the expression of such enzymes in wild-type strain. Inducing gene expression in proteins involved in ethanol production at high temperatures was also evaluated in other microorganisms. High expression of genes encoding hexokinase, glyceraldehyde-3-phosphate dehydrogenase, alcohol dehydrogenase and, isocitrate dehydrogenase in *S. cerevisiae*, and high expression of *adh* and *tdh2* genes in *P. kudriavzevii* are some of the examples [56, 60]. Accordingly, it appears that high-temperature stress conditions may negatively affect glycolysis, TCA, and PE pathways, which play important roles in metabolism, growth, and bioethanol production. Therefore, as a response mechanism, expression of genes related to these metabolic processes enhances and controls bioethanol synthesis under heat shock and also helps the cell to produce sufficient energy needed to restore destroyed proteins and macromolecules [62]. On the other hand, the accumulation of sugars and carbohydrates confer thermotolerance to cells in some cases. For example, accumulation of sorbitol in *Z. mobilis* or trehalose in *S. cerevisiae* and *P. kudriavzevii* results in the protection of proteins from denaturation, destruction, and aggregation [30, 56, 60]. The main physiological changes under high-temperature stress conditions in *Z. mobilis*, *S. cerevisiae*, and *P. kudriavzevii* are listed and compared in Table 1.

In general, *Z. mobilis* can maintain cell activity and overcome the adverse effects of high temperature by altering physiological properties, biosynthesizing membrane proteins and transporters to improve stability, producing metabolites and proteins to restore and protect intracellular structures.

### High glucose and osmotic stress condition

In most batch fermentation processes, the optimal sugar concentration is about 190 g/l, and sugar concentration greater than 500 g/l can lead to a stress condition that results in substrate inhibition and reduces bioethanol production [63]. Cellular metabolism is reduced under such stress conditions, and the enzymes responsible for glucose breakdown are inhibited [64]. When cells are exposed to hyperosmotic extracellular stress, they generally employ two strategies to overcome the negative effects. First, the accumulation of osmolytes such as polyols, sugars, betaines, and ectoins, also known as compatible solutes, and second, osmoadaptation to the saline cytoplasm, in which an efflux of ions such as K^+^, Na^+^, and Cl^−^ is transported into the cell and accumulates in the cytoplasm [64]. Studies on *Z. mobilis* have shown that both strategies are used to overcome such stress conditions. Two main effects of osmotic stress on *Z. mobilis* are discussed below.

#### Effects on cell membrane composition and transporters

According to studies on membrane transporters, 30 genes encoding membrane proteins are differentially expressed under osmotic pressure, including the clay transporter system, ABC proteins, the type I secretion system, sugar transporters, and heavy metal transporters [65]. TolC type I protein is a significant protein that is highly expressed under osmotic stress conditions. In *Z. mobilis*, this protein was highly expressed under osmotic stress conditions, although its function in this species has not been elucidated comprehensively [65]. TonB-dependent transporters (TBDT) is another membrane protein inclusively expressed under osmotic stress conditions in *Z. mobilis* [65]. This outer membrane protein is responsible for siderophore transportation, vitamin B12, nickel compounds, and carbohydrates [66]. Under normal conditions, this protein is not expressed by *Z. mobilis*, whereas its expression is increased in *Z. mobilis* at high glucose concentration. Although, although the function of this protein in *Z. mobilis* is still unknown, its high expression may contribute to osmotic stress tolerance [65]. In addition, transmembrane proteins such as heavy metal transporters, Mg^2+^ transporter proteins, and chloride channel proteins are upregulated [65] is consistent with this phenomenon that efflux of ions inside the cytoplasm will be increased in response to high glucose concentration. Therefore, changes in cell membrane protein structure are a mechanism that is used to cope with the negative effect of hyper-osmotic conditions. This phenomenon is also observed in other ethanologenic yeasts such as *S. cerevisiae* and *K. marxianus* [67, 68].

#### Effects on growth rate and metabolism

*Z. mobilis* is able to grow at a glucose concentration of 10 to 15% [30]. In a study performed by Sturch et al., the result revealed that by enhancing initial glucose concentration in the media from 20 g/l to 200 g/l, specific bacterial growth rate and cell yields will be decreased, whereas lag-time of growth will be increased significantly [65]. Wild type of *Z. mobilis* can produce ethanol from initial sources of glucose, fructose, and sucrose through the ED pathway by the activity of two enzymes of pyruvate decarboxylase (PDC) and alcohol dehydrogenase (ADH). Under osmotic stress conditions, the expression of these two enzymes will be decreased significantly, which also clearly explains the reduction of ethanol production by *Z. mobilis* [69]. One of the key enzymes involved in osmoadaptation in *Z. mobilis* is the enzyme glucose-fructose oxidoreductase (GFOR), which is encoded by the *gfo* gene. This periplasmic enzyme accounts for approximately 1% of the soluble protein in the cell [30]. In addition, this enzyme can convert glucose and fructose to gluconolactone and sorbitol, respectively [70].

**Fig. 2:**
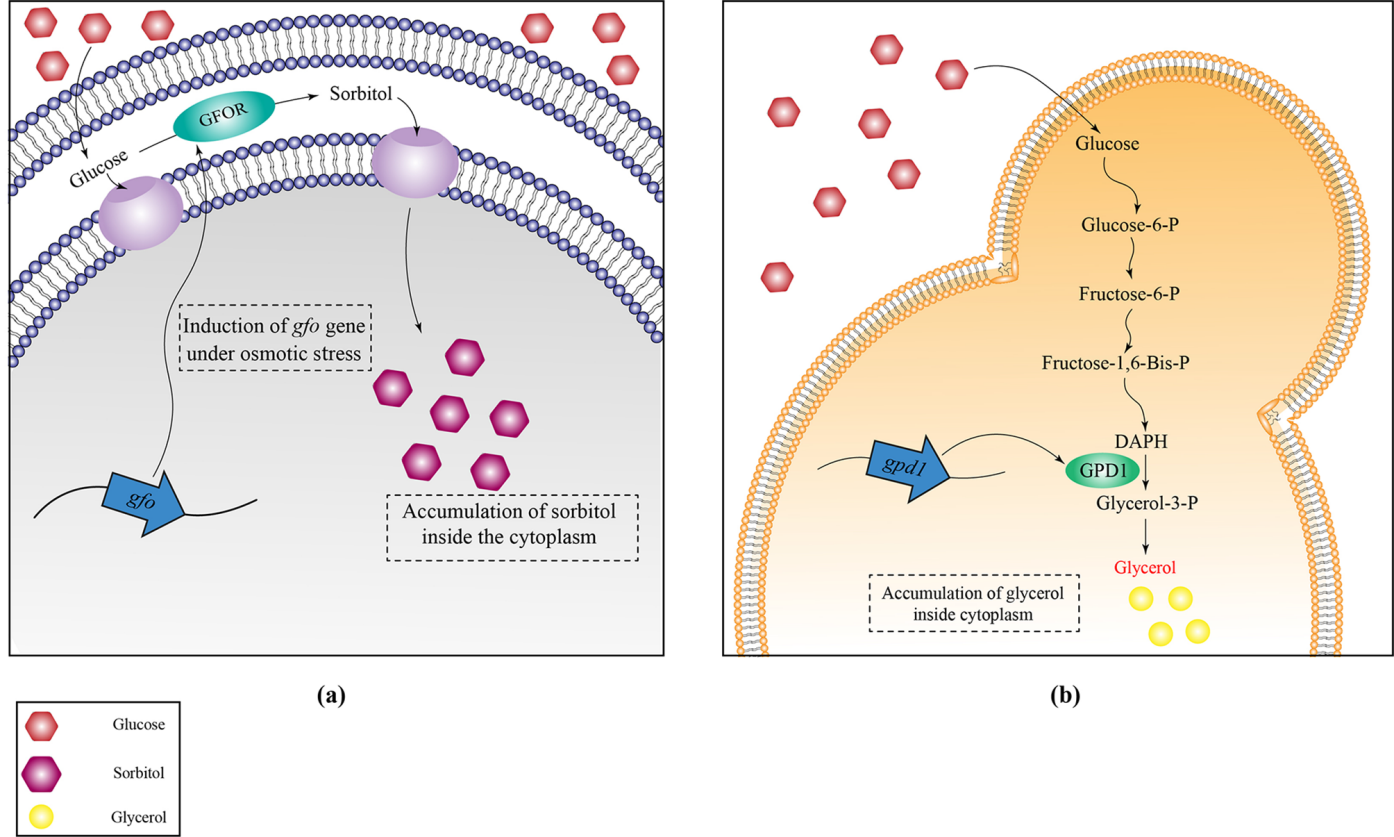
Comparing compatible solute production mechanism in***Z. mobilis *and *S. cerevisiae *during high glucose and osmotic stress conditions.** In *Z. mobilis* (**a**), expression of *gfo* gene encodes glucose-fructose oxidoreductase, and in *S. cerevisiae* (**b**), *gpd1* gene encodes glycerol-3-phosphate dehydrogenase contributes to tolerance to high glucose concentrations

Sorbitol is one of the compatible solutes whose accumulation in the cell increases the specific growth rate and volumetric ethanol production due to the positive effect of this compound on the expression level of the *pdc* and *adh* genes [30, 34]. Although more studies still needed to be done to understand the molecular effect of sorbitol, studies indicated that the presence of sorbitol inside the cell would stabilize the cell membrane and recover the cell fluidity under osmotic stress conditions [69]. In addition, under conditions of osmotic stress, reactive oxygen species can be generated that can damage DNA, cell membrane lipids, and especially structural and functional proteins [65]. Comparison of protein synthesis under osmotic stress conditions in the presence and absence of sorbitol revealed that this sugar plays a significant role in protein synthesis and protection [69]. The accumulation of compatible solutes is also observed in other ethanologenic species such as *S. cerevisiae* and *K. marxianus*. In these two microorganisms, the accumulation of glycerol leads to osmoadaptation [67]. Interestingly, comparison of the production of compatible solutes in *Z. mobilis*, *K. marxianus*, and *S. cerevisiae* revealed that the production of sorbitol and glycerol is an enzymatic process. As shown in Fig. 2, the production of sorbitol in *Z. mobilis* occurs through the activity of the GFOR enzyme, and in *S. cerevisiae* the production of glycerol is controlled by the *gpd1* gene, which encodes glycerol-3-phosphate dehydrogenase [68, 71]. It can be inferred that in *Z. mobilis*, exerting several mechanisms from membrane remodeling to substrate accumulation give rise to its osmoadaptation ability.

### Effect of stress conditions on expression of regulatory proteins in *Z. mobilis*

#### Regulatory network in *Z. mobilis*

Under stress conditions, different strains employ a variety of regulatory networks to cope with external stress conditions. These regulatory networks consist of three main components: Transcription factors, sigma factors, and regulatory RNAs [72]. Table 1 summarizes studies conducted on key regulatory networks activated during the four major fermentation stress conditions (ethanol, lignocellulose, temperature, and osmotic stress) in *Z. mobilis* and compares them with other ethanol-producing species.

#### Transcription factors

Several transcription factors control gene expression in microorganisms, and can respond to different stress conditions by regulating gene expression about 54 repressors and activators have been found in the genome of *Z. mobilis* [73]. Lrp/AsnC family, Xre family, LysR family, LytR family, TetR family, MarR family, RpiR family, Psp family, GntR family, and LacI are some of the major transcriptional regulators expressed in *Z. mobilis* under a variety of stress conditions (Table 2) [21, 36, 65].

**Table 2:** Transcription factors and their role in cell activity and performance during bioethanol fermentation stress conditions in *Z. mobilis*

Transcription Factors	Gene	Function	Stress condition during bioethanol production in Z. mobilis	Similarity to E. coli	References
Lrp/Asc family	*lrp*	Amino acid metabolism, small molecule transport, central carbon metabolis, transportation of small peptides	Low level in ethanol and lignocellulose stress, High level in osmotic stress	60%	[24, 36, 82]
Xre family	*xre*	Regulate high temperature and oxidative stresses, control cell metabolism like nitrogen metabolism, response DNA damage stresses	Down-regulate under Ethanol stress, Up-regulate under osmotic stress	85%	[21, 131]
LysR family	*lysR*	Regulate cell metabolism (carbohydrate metabolism), motility, cell division, oxidative stress, nitrogen fixation, quorum sensing, expression of transporters	Ethanol, lignocellulosic inhibitors, and osmotic stress	46%	[21, 36, 81]
TetR family	*tftR*	Regulating expression of efflux proteins such as ABC transporters, fatty acid biosynthesis, osmotic stress, cell division by controlling FtsZ formation	Ethanol and furfural stress	48%	[21, 36, 80]
MarR family	*marR*	Metabolic pathway such as catabolism of phenolic compounds, virulence, and stress responses such as expression of multi drug efflux pumps	Downregulate under Ethanol stress	63%	[21, 132, 133]
RpiR family	*ripr*	Regulate catabolism of sugars,	Ethanol stress	22%	[21, 134]
Psp family	*pspA*	Regulate expression of *psp* operon under stress conditions, down regulate motility under stress condition, recover cell envelope under stress condition	Ethanol, Lignocellulosic inhibitors, and hyperosmotic stress condition	66%	[21, 36, 65, 135, 136]
	*pspB*			No significant similarity	
	*pspC*			70%	
	*pspF*			68%	
GntR family	*gntR*	ABC transporter, antibiotic stress response	Furfural stress	52%	[36, 135]
LacI family	*lacI*	Regulate metabolic pathways, Carbon catabolism	Furfural stress	53%	[36, 137]
LytR family	*lytR*	Control activity of autolysin enzymes, maintain cell wall structure, control cell growth in bacteria	Furfural stress	54%	[36, 138]

#### Sigma factors

Sigma factors are another group of proteins that play an important role in the regulatory system [74–76]. Since the sigma factors are almost completely characterized in *E. coli*, comparison was made with *Z. mobilis.* Seven sigma factor proteins have been discovered in *E. coli*, including sigma 70, sigma 54, sigma 38, sigma 32, sigma 28, sigma 24, and sigma 19 [42, 76]. According to analyses the analyses compiled in Table 3, the sigma factor proteins in *Z. mobilis*, including sigma-D, sigma-N, sigma-H, sigma-F, and sigma-E, have structural similarity of 62%, 35%, 40%, 36%, and 30%, respectively, with the to sigma factors in *E. coli*. Compared to sigma factors in *Z. mobilis*, only five homologous proteins have been observed in this species. Sigma 38 and sigma 19 have not been detected in *Z. mobilis* species [44]. In *E. coli*, and sigma factor H, sigma factor E, sigma factor N are activated under heat shock stress conditions [76]. These regulatory proteins are activated in *E. coli* for the same purpose as in *Z. mobilis* with the same function. For example, sigma-H and sigma-E are activated during heat shock to control protein folding in the cytoplasm and periplasmic region, respectively [76]. Furthermore, activation of Sigma-N at high temperatures leads to induction of PspF and stabilization of the cell membrane in *E. coli* [70]. However, sigma-N has the same function (PspF activation) in *Z. mobilis* and *E. coli*, this protein role in ethanol and osmotic stress response in *Z. mobilis*. Apart from Sigma-N’s significant role in both *Z. mobilis* and *E. coli*, the two species also exhibit significant similarity (> 60%) in their Psp transcription factors, as described in Table 3. Therefore, PspA and PspF appear to stabilize the membrane structure under stress conditions and induce the *psp* operon in both species. In Table 3, the general characteristic of sigma factors in *Z. mobilis* and their similarity to the same proteins in *E. coli* are summarized.

**Table 3:** Sigma factors and their role in cell activity and performance during bioethanol fermentation stress conditions in *Z. mobilis*

Sigma Factors	Gene	Function	Stress condition during bioethanol production in Z. mobilis	Similarity to E. coli	References
Sigma-70 (sigma-D)	*rpoD*	Housekeeping sigma	Furfural and ethanol stress	62%	[21, 36, 61, 76, 92]
Sigma-54 (Sigma-N)	*rpoN*	Nitrogen regulation, psp opern regulation	Ethanol and osmotic stress	35%	[21, 55, 59, 70]
Sigma-32 (Sigma-H)	*rpoH*	Heat shock (cytoplasm)	Heat shock and hyperosmotic stress condition	40%	[61, 76]
Sigma-28 (Sigma-F)	*fliA*	Flagellar proteins	Ethanol and furfural stress	36%	[21, 36, 55, 70]
Sigma-24 (Sigma-E)	*rpoE*	Heat shock (periplasm)	Extreme Heat shock and ethanol stress	30%	[21, 55, 70]

#### Regulatory RNAs

Regulatory small RNAs also play an important role in modulating gene expression under various stress conditions such as ethanol, temperature, pH, and oxidative stress [77]. These regulatory compounds maintain mRNA stability and translation [78]. In addition, they fulfil their regulatory function and regulate gene expression by base-pairing with the 5´ UTR region of mRNA [78]. In *Z. mobilis*, fifteen sRNAs known as Zms have been discovered in which Zms2, Zms4, Zms6, and Zms18 seem to play a vital role in response to different stress conditions [34, 81]. In *Z. mobilis*, the 5´UTR region was found to control gene expression of the RNA chaperone Hfq under ethanol stress.

Therefore, the UTR region controls cell performance under ethanol stress conditions by up and down- regulating genes expressing Hfq proteins, thioredoxin reductase, etc.; however, no significant UTR regulatory activity was detected in *Z. mobilis* under xylose and acetate stress [79]. In the following section, the role of each protein during stress response in *Z. mobilis* will be discussed.

### Regulatory networks during bioethanol fermentation stress condition

#### Ethanol stress condition

As we have already discussed, ethanol stress has some effects on the physiological structure of the cell, including changes in membrane proteins, ethanol production, growth rate, DNA replication, and membrane permeability [20, 21]. These changes are controlled by the regulatory system that controls gene expression under such environmental conditions. Transcriptome analysis in *Z. mobilis* revealed that the expression of 33 transcription factors decreased under ethanol stress conditions, including MarR, XRE, and HxIR, whose expression changed 1.9, 1.5, and 1.8 fold respectively under high ethanol concentrations [21]. TetR family transcriptional regulators are another group of the regulatory system that controls gene expression. These regulatory factors have a variety of roles, such as controlling metabolism, antibiotic resistance, and other physiological changes in the cell [80]. LysR and RpiR are also transcriptional factors regulating genes involved in quorum sensing, motility, and carbon metabolism [81]. Transcriptome analysis of *Z. mobilis* showed that these three factors (TetR, LysR, and RpiR) are abundant under ethanol stress situations [21]. Considering that these factors are upregulated under ethanol stress and the role of transcription factors, it is reasonable to assume that high expression of these proteins induces the expression of ABC transporters and increases sugar degradation, regulates cell metabolism, and stimulates fatty acid synthesis to compensate for the deleterious effects of ethanol on the physiological aspect of *Z mobilis*.

In addition, there were two genes, ZMO1107 and ZMO0347, whose expression significantly decreased in *Z. mobilis* under ethanol stress [21]. Analysis and homologous study on these two genes revealed that they have approximately 50% and 70% identity to Lrp/AsnC family and Hfq chaperone proteins in *E. coli*, respectively. In most bacterial strains, Lrp proteins are responsible for the metabolism of amino acids; for instance, in *E. coli*, its’ role is in the catabolism of alanine amino acid [82]. Compared to ethanologenic yeasts such as *K. marxianus*, metabolism of amino acids under ethanol stress is a critical activity that occurs under the control of the transcription factor GCN4 [26]. GCN4 is a type of transcription factor that is widely expressed in yeast cells [83]. This transcription factor is the main central domain for protein and amino acid biosynthesis, especially under stress conditions [26].

An important factor regulating gene expression under stress conditions, particularly heat shock and ethanol stress, is heat shock protein (Hsp). Hsps are functionally divided into two groups. Some Hsps, including GroEL, DnaK, GrpE, and DnaJ, are chaperones that attach to misfolded proteins and change their folding properly when the cell is exposed to temperature stress conditions. Other Hsps function as proteases such as Clp, Lon, and FtsH, which are responsible for removing and denaturing misfolded proteins before cell dysfunction occurs [84]. Genomic analysis of *Z. mobilis* revealed several complete ORFs for these Hsps from different chaperones to various ATP-dependent proteases. These proteins will be up-regulated in stress conditions like ethanol and heat shocks in *Z. mobilis* to combat misfold protein aggregation in the cell [73]. In an investigation by Thanonkeo et al., the expression of two Hsp proteins under ethanol concentration from 3.5 to 14% was studied in *Z. mobilis*. The comparison of *groEL* and *groES* expression between stressed and non-stressed cells suggested that the expression of these two proteins increased about three to six-fold under ethanol stress conditions [85]. However, according to studies by He et al., no significant change was observed in the expression of molecular chaperones including DnaJ, DnaK, Hsp70, GroES, GroEL, and Hsp33 in *Z. mobilis* [21].

It appears that these proteins are necessary for the enhancement of ethanol tolerance in other ethanologenic species such as *K. marxianus* and *S. cerevisiae*, either [25, 26, 86]. In *K. marxianus*, transcriptome analysis showed three Hsp proteins, including Hsp60, Hsp26, and Hsp78, significantly upregulate under ethanol stress conditions [26]. Different Hsp proteins were also observed in *S. cerevisiae* under ethanol stress, including Hsp104, Hsp82, Hsp70, Hsp12, Hsp90, etc [19, 25, 87]. . . Ethanol stress may have some negative effects on cellular components and denature proteins; consequently, upregulation of these proteins may refold proteins and prevent aggregation of misfolded proteins within the cell [25, 26, 86].

Genomic analysis of *Z. mobilis* revealed that this microorganism is able to express another transcription protein called Psp family [73]. Studies by He et al. revealed that three types of Psp proteins, including PspA (1.64-fold change), PspB, and PspC showed differential expression under ethanol stress [21], so it is suggested that under ethanol stress conditions, these Psp proteins activate some other proteins that help the cell to recover cell membrane and alleviate the negative effect of ethanol on cell physiology.

Sigma factors were also upregulated under ethanol stress conditions. According to studies, sigma E (1.3-fold), rpoD (1.7-fold), rpoN (1.2-fold), fliA (1.4-fold) were differentially expressed, suggesting that their expression is critical for maintaining cell survival under stress conditions [21]. Upregulation of rpoD is consistent with the expression of Ton-B transporters. According to studies, this transporter regulates under the control of ECF sigma factors, a protein in the group of sigma-70 that regulates the expression of periplasmic and outer membrane proteins [66]. According to recent studies on the role of sigma factors in *Z. mobilis*, sigma32 and sigma24 do not appear to be responsible for the response to high ethanol concentrations [61]. Homology and sequence analysis of sigma factor proteins in *Z. mobilis* is compared with *E. coli* in Table 3.

**Fig. 3:**
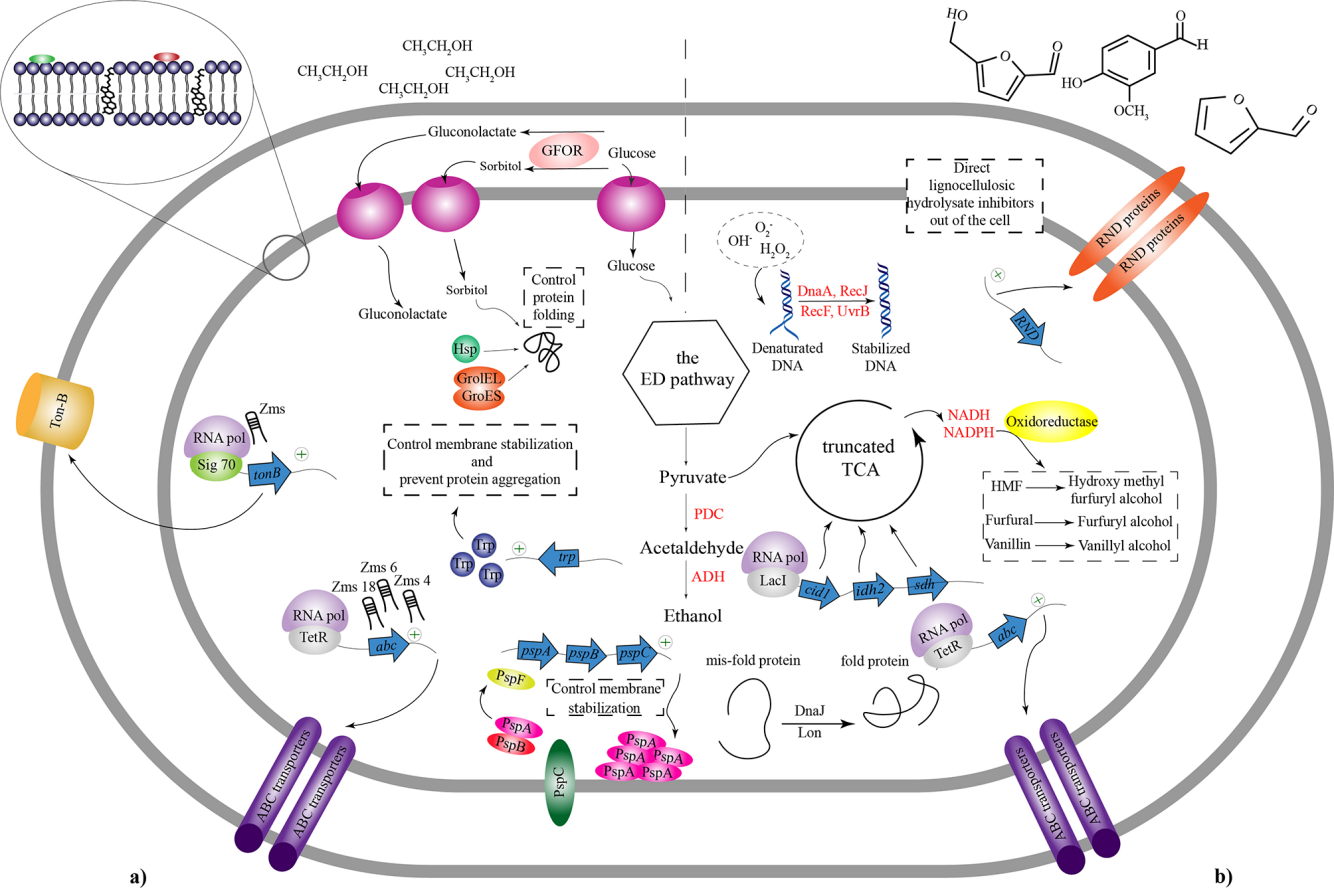
Physiological and molecular stress responses in*Z. mobilis* during ethanol and lignocellulosic inhibitor stress conditions. **a) Ethanol stress**: Ethanol stress induces changes in the lipid membrane composition of *Z. mobilis* strains, resulting in a decrease in the level of unsaturated fatty acids to saturated fatty acids. During normal conditions, PspF binds to PspA protein. However, during ethanol stress, this protein is separated, leading to increased expression of the psp operon and high levels of PspA production. PspA proteins attach to the cell membrane structure, maintaining membrane structure and stability. Furthermore, during ethanol stress, the GFOR enzyme converts glucose to gluconolactate and sorbitol. The accumulation of sorbitol, as well as Hsp proteins and GroEL/GroES chaperones, controls protein conformation and folding. Interestingly, increasing the expression of tryptophan operon increases the level of this amino acid in the cytoplasm, preventing protein accumulation inside the cell. Additionally, the activity of regulatory RNAs (Zms), transcription factors such as TetR and sigma-70 enhance the expression of membrane transporters (ABC transporters and Ton-B), which significantly control membrane permeability under ethanol stress **b) Lignocellulosic inhibitor stress**: Under stress conditions, reactive oxygen species (ROS) such as OH^−^, O^2−^, and H_2_O_2_ may be produced, which can denature DNA structure. In response, DNA repair systems and proteins such as RecJ, DnaA, RecF, and UvrB are activated to maintain DNA conformation and stability. Regulatory networks such as LacI and sigma-70 increase the expression of genes that regulate TCA enzymes, leading to increased NADH and NADPH levels. Together with oxidoreductase enzymes, these cofactors convert lignocellulosic inhibitors such as furfural, HMF, and vanillin into low-toxic structures. The activity of Lon and DnaJ proteins helps retain protein structure and fold. Transcription factors such as TetR increase the expression of transmembrane proteins such as ABC transporters and RND, which inhibit the negative effects of lignocellulosic inhibitors and direct them outside the cell

Besides proteins that can act as regulatory elements, there are also RNAs that can be involved in regulatory processes, which are also more efficient than transcription proteins because they do not need to be translated and directly bind to mRNAs [78]. Studies on such small RNAs in *Z. mobilis* revealed that four regulatory RNAs have significant functions in gene expression. Three sRNAs (Zms2, Zms6, and Zms18) showed remarkable differential expression under different ethanol concentrations from 0 to 5% [34]. Moreover, other experiments on the role of sRNA in response to ethanol stress showed that, in addition to Zms6, Zms4 is also among vital regulatory elements in *Z. mobilis* [88]. These regulatory RNAs participate in different cell functions such as expression of ABC transporters, Ton B receptors, carbohydrate utilization, and cell motility [34, 88]. Besides small RNA that was previously studied, some regulatory regions in 5´ untranslated regions control the expression of genes in stress conditions. In a study by Cho et al., they used transcriptomic data to analyze and identify the 5´UTR regions in *Z. mobilis*. The results suggest that there are 101 UTR regions in the genome of these species that act as a regulatory modules. Of all these untranslated regions, 36 UTRs function as regulators in stress responses. To test this concept, they constructed 36 5´UTR- GFP libraries and analyzed the gene expression of GFP proteins under stress and standard conditions (a strain without 5´UTR region used as control). The results showed that two strains containing the 5′UTR of RNA binding protein Hfq and the 5′UTR of thioredoxin reductase had significant fluorescent changes under 5% ethanol concentration compared to control. Furthermore, this experiment was also performed under xylose and acetate stresses in which the result showed no significant changes in regulatory regions of strain that contain the 5′UTR of thioredoxin reductase [79]. In summary, in *Z. mobilis*, ethanol stress leads to some physiological changes in which the expression of transcription factors such as TetR, Psp, and RpiR helps the cell to regulate cell metabolism, membrane viability, fatty acid biosynthesis, and various transporters. Along with transcription factors, sRNAs including Zms2, Zms4, Zms6, and Zms18, by controlling the regulation of ABC transporters, carbon utilization, and cell motility, are another group of regulators that their expression is critical for cell performance under such stress environment. The overall physiological and molecular responses in *Z. mobilis* during ethanol stress are summarized in Fig. 3(a).

### Lignocellulosic hydrolysate inhibitor stress condition

Lignocellulose inhibitors resulting from hydrolysis activity also affect the regulation of various genes and proteins, leading to bacterial tolerance to such stress conditions. Transcriptome profiling of *Z.mobilis* under furfural stress response showed that some proteins are upregulated, and some are downregulated as a stress response mechanism. Under furfural stress response, chaperone proteins like DnaJ and proteases like Lon gene remarkably upregulated, while groEL- groES did not show significant changes in their regulation [36]. Moreover, upregulation of transcriptional regulators such as LysR, LytR, GntR, TetR, LacI was also observed under furfural stress conditions in *Z. mobilis* [36]. LytR is a regulatory protein in the LytTR two-component response regulators that control cell wall structure and function and bacterial growth under stress conditions. This protein in *Z. mobilis* has approximately 54% similarity to Skn7 protein in *S. cerevisiae*, according to blastp analysis. This protein is also part of a two-component system called SLN1-YPD1-SKN7 in eukaryotic cells that serves as a transcription factor in signaling pathways under osmotic and oxidative stress conditions. Interestingly, this protein plays a central role in controlling cell wall structure and integrity during external environmental stress in *S. cerevisiae* [89].

Another factor that significantly affects the regulation of genes under stress conditions is Hfq, a global regulatory system that acts as an RNA-binding protein. Hfq is an RNA-binding protein that regulates gene expression during post-transcriptional levels. Hfq plays various functions in the RNA metabolism of bacteria, including stabilizing small non-coding RNAs (sRNAs) and aiding in their interactions with messenger RNAs (mRNAs), which consequently impact the stability and translation of the specific targeted mRNAs [90]. This protein is homologous to Lms proteins found in eukaryotic cells in both functions and structures. Studies showed that this protein has a significant role in resistance to lignocellulosic inhibitors such as furfural, HMF, acids, and ethanol [91]. In an experiment by Yang et al., they use *Z. mobilis* and its mutant to determine the effect of byproducts such as furfural, vanillin, and HMF produced under the fermentation process on the growth rate and production of ethanol by the microorganism. The results showed that the mutant, which has functional Hfq, has a shorter lag phase and high cell density than the wild type. These results assumed that Hfq is an essential protein for optimal growth of *Z. mobilis* under such stress conditions [91].

The genome sequence of *Z. mobilis* revealed that it has rpoH (sigma-32 factor), rpoE (sigma-24 factor), rpoN (sigma-54 factor), rpoD, rpoA, and fliA (sigma F), but microarray analysis revealed that only rpoD and fliA are differentially expressed under furfural stress conditions [36]. Sigma 70 is another regulatory factor that helps RNA polymerase recognize the transcription site and promotors encoded by the *rpoD* gene. The effect of rpoD on the growth and performance of bacteria under furfural stress was investigated by comparing control strains and mutant strains (ZM4-rpoD) [92]. The results determined that increased furfural concentration under growth profile will also increase in mutant strains. The importance of this protein on the cell under stress conditions relies on its function, which is controlling and regulating gene expression. Thus, this protein promotes the expression of genes involved in metabolic activity, enabling the cell to cope with stressful situations [92].

In addition, the effect of two regulatory factors, SigE and Hfq, was studied under furfural and acetic stress conditions. The comparison of two recombinant strains that highly expressed Sigma E and Hfq proteins with the wild type revealed that these two factors significantly affect the tolerance of *Z. mobilis* to stress conditions [93]. However, the increase in furfural and acetic acid concentration in the medium reduced growth rate and ethanol production in all strains, but the recombinant strain was less affected. Moreover, comparing two recombinant strains revealed that ZM4- sigE has more ethanol production and growth rate than ZM4-hfq recombinant strain [93].

In general, under LCH inhibitors stress conditions in *Z. mobilis*, sigma factors such as fliA and sigma D along with Hfq proteins are among important proteins regulating gene expression in bacteria. Furthermore, upregulation of transcription factors such as TetR, LacI, GntR, LysR, and LytR by regulating the expression of ABC transporters, Na^+^/H^+^ transporters, cell division, bacterial growth, and cell membrane structure helps the cell to compensate for the negative effect of LCH inhibitors stresses during bioethanol production on bacterial growth and function. The physiological and molecular stress response during LCH inhibitors stress conditions and the regulatory networks activate under such stress conditions is illustrated in Fig. 3(b).

#### High temperature stress condition

The molecular response of *Z. mobilis* to different temperatures is complex and involves the expression of genes control protein quality, membrane stability, translation, transferring proteins, and DNA repair system [44]. The heat shock proteins GroEL, GroES, and mHsp70 are among the most abundant proteins in the cell under heat stress [44]. Investigations on the expression of these proteins under heat shock in *Z. mobilis* revealed that the expression of *groEL* and *groES* had been increased about three to six-fold under heat shock stress compared with non-stressed cells [44].

In conclusion, Hsp proteins are among critical factors that their expression can protect peptidoglycan and prevent protein denaturation under high temperatures. Besides heat shock proteins, other proteins such as sigma factors play a crucial role in adaptation to heat shock stresses. Among several sigma factors sequenced in *Z. mobilis* genome, sigma 24 has a significant role in responses to heat shock and high temperatures [61]. In the study performed by Benoliel et al., results determined that sigma factor 32 and 24 regulates gene expression in both heat shock and osmotic stress in *Z. mobilis* [61].

According to transcriptome analysis in *Z. mobilis*, no significant changes in the expression of transcription factors were observed under high temperature, except WrbA protein, a regulatory protein that binds to tryptophan inhibitors (TrpR) and controls the expression of this amino acid [52]. Moreover, proteins that control protein quality, such as M16 peptidase (Zn- dependent protein) and DegP protein, a periplasmic serine peptidase, were also differentially expressed [52]. These proteins remove misfolded proteins in the periplasmic region to control the negative impact of their accumulation on the cell membrane under heat shocks [94].

**Fig. 4:**
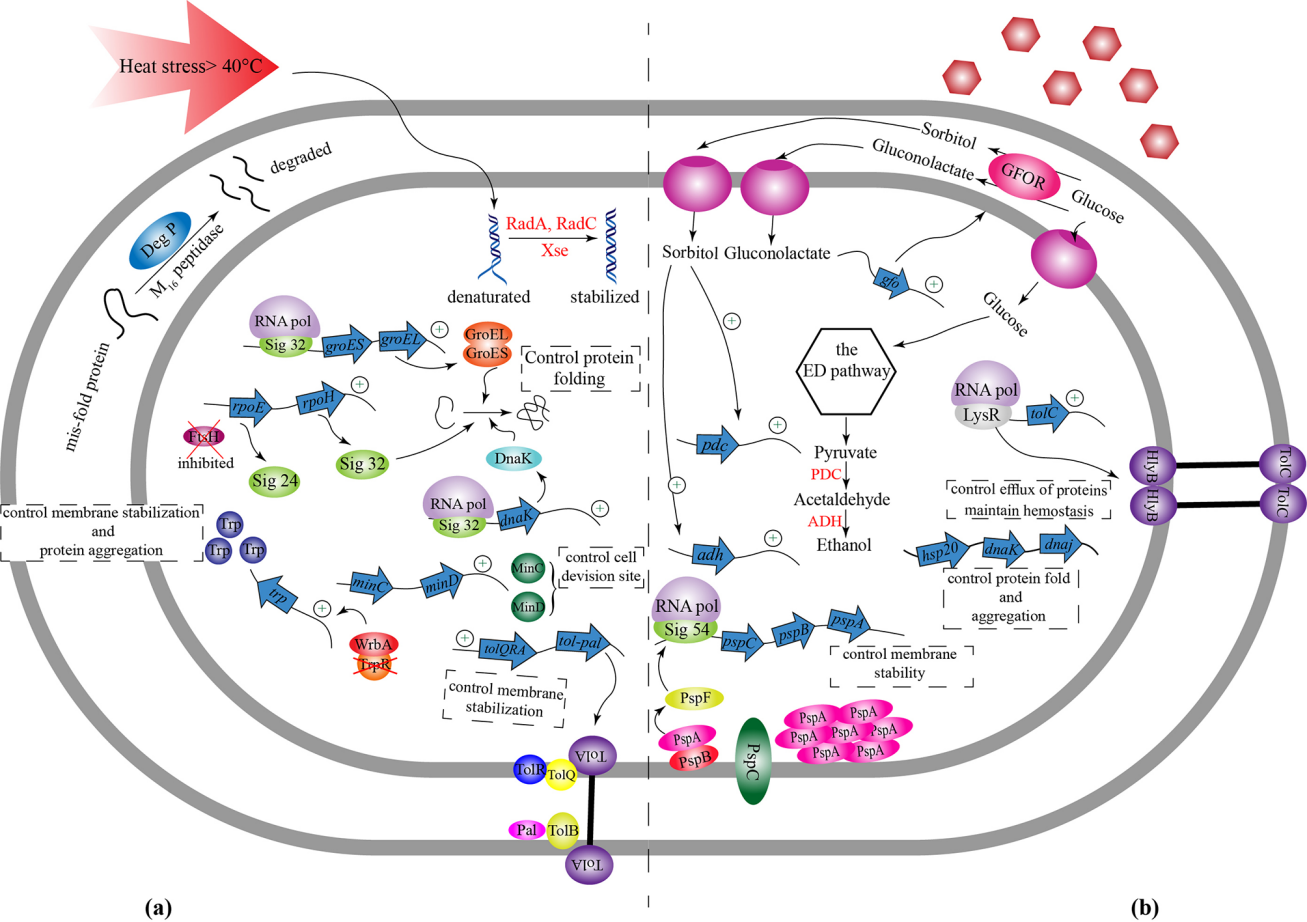
Physiological and molecular stress responses in***Z. mobilis* during high temperature and high glucose stress conditions. a) High-temperature stress**: In *Z. mobilis*, high temperatures can negatively affect its cell performanceThe elevated temperature can disrupt DNA structure by breaking hydrogen bonds between nucleobases, resulting in denaturation. To counteract this, the DNA repair system is activated, aided by proteins such as RadA, RadC, and Xse, to stabilize and refold the DNA. Conversely, FtsH is inhibited during this stress condition. This protein typically binds to Sigma-32 to inhibit its activity and expression under normal conditions. However, in high-temperature stress conditions, FtsH inhibition activates Sigma-32, which, in conjunction with other chaperones and proteins, refolds denatured proteins and stabilizes their structure. Notably, WrbA protein binds to TrpR (the trp operon inhibitor) to increase tryptophan expression, preventing protein aggregation within the cytoplasm. In the periplasmic region, DegP and M16 proteins degrade misfolded proteins to prevent aggregation within the cell. Finally, increased expression of transmembrane proteins and MinC and MinD proteins helps regulate membrane structure and cell division under high-temperature stress, respectively **b) High glucose stress**: Elevating the concentration of glucose in the extracellular environment induces a series of intracellular signals that ultimately upregulate the expression of genes, such as the *gfo* gene. The increased activity of the GFOR enzyme facilitates the conversion of extracellular glucose into sorbitol. The accumulation of sorbitol inside the cell, in conjunction with chaperones like DnaJ, DnaK, and Hsp20, helps regulate and refold denatured proteins. Moreover, sorbitol enhances the expression of PDC and ADH enzymes, which control bioethanol production. Additionally, the transcription factors Sigma-54 and PspF increase the expression of the psp operon, promoting membrane stabilization during glucose stress. Transmembrane proteins, including TolC, play a crucial role in hemostasis by facilitating the flow of proteins and sugars outside the cell

Compared to *S. cerevisiae*, according to a study by Kim et al., profiling transcription regulators that are expressed at temperatures above 40℃ revealed that factors such as Hsf1, Msn2, Msn4, and Yap1 were significantly increased in thermotolerant strain, while the wild strain did not have any changes in these kinds of proteins [56]. These regulatory factors are summarized and compared in Table 1 in *Z. mobilis*, *S. cerevisiae*, and *P. kudriavzevii*. In general, it appears that under high-temperature stress in *Z. mobilis*, proteins quality control system and Hsp proteins are the essential elements for controlling cell tolerance to such stress conditions (Fig. 4 (a)).

#### High glucose and osmotic stress condition

During osmotic stress, transcriptome analysis in *Z. mobilis* revealed some changes and upregulations in the expression of factors and proteins, including RNA polymerase sigma factor 32, sigma 54-specific transcriptional activator PspF, and catalase [65]. According to studies on *Z. mobilis*, heat shock proteins such as Hsp20, heat shock protein DnaJ domain-containing protein, and chaperone DnaJ domain-containing protein were upregulated in hyperosmotic stress conditions [65]. Sigma 54, which regulates *psp* operon, was also upregulated, resulting in the upregulation of two major Psp proteins, PspA and PspB [65]. High expression of the Psp family transcription factor was also observed during ethanol stress. This transcription factor may play a critical role during the bioethanol fermentation process and act as a regulatory protein in response to various stresses. In contrast, other sigma factors showed a significant decrease in expression during osmotic stress conditions. Sigma 70 is a vital sigma factor that plays an essential role in cell growth under standard conditions. The species that expressed sigma 70 under high glucose concentration have lower cell densities because the increased expression of sigma 70 may result in a decreased expression of transporter genes and osmoprotectants production such as sorbitol which may result in a slow adaptation to osmotic shocks [61]. Comparing Hsp production with other species, different Hsp proteins, including Hsp26, Hsp42, Hsp31, Hsp82, and Hsp12, were significantly changed in hyperosmotic stress conditions in ethanologenic yeasts such as *K. marxianus* and *S. cerevisiae* (Table 1) [68, 95].

In summary, upregulation of sigma factors in *Z. mobilis* under hyperosmotic stress conditions regulates the expression of heat shock proteins to degrade misfolded and unfolded proteins. Expression of sigma 54 leads to the regulation of Psp proteins, a group of regulatory proteins that control cell envelope structure. Finally, regulation of transporters and efflux proteins, carbohydrate metabolism, and bacterial cell growth are affected by the expression and activation of transcriptional regulators such as LysR, AsnC, and Xre under hyperosmotic conditions Fig. 4(b) presents all regulatory factors and proteins that contribute to the response mechanism to osmotic fermentation condition in *Z. mobilis*.

During bioethanol production, cells may simultaneously encounter multiple types of stress. These stressors can have antagonistic or synergistic effects on microbial cells. A study on the cellular responses of *S. cerevisiae* to high temperature and osmotic stress revealed that heat, acetic acid, ethanol, and oxidative stressors resulted in synergistic adverse effects, which consequently reduced the growth, ethanol production rate, and long-term thermal stability of *S. cerevisiae* and ethanol productivity at high temperatures [96]. Another investigation of the synergistic effect of temperature and ethanol on *S. cerevisiae* revealed that fermentation temperature and high ethanol concentration influenced the dynamic behavior of *S. cerevisiae*, leading to variations in biomass, ethanol, and glycerol production [97]. The combined effects of cold, acid, and ethanol stress on the membrane stability of *Oenococcus oeni* were investigated. The results indicated that ethanol and acid stress had severe effects on cell viability and increased membrane fluidity, whereas cold shock reduced the deleterious effects of these two stress conditions on the cell and maintained cell viability [98]. Moreover, changes in cell viability and membrane structure were observed in *E. coli* during a combination of temperature and osmotic stress [99].

In general, physiological responses in *Z. mobilis* during ethanol fermentation stress conditions include several changes, including changes in cell membrane structure, such as (1) changes in membrane proteins and transporters, as well as phospholipid structure to maintain membrane stability; (2) activation of the DNA repair system that alleviates the negative effects of ROS accumulation inside the cell and their influence on DNA structure; (3) activation of proteins such as heat shock proteins that degrade and refold misfolded proteins; and (4) accumulation of sugars (sorbitol) and amino acids (tryptophan) inside the cell to stabilize the cell membrane and prevent protein aggregation in the cytoplasm. In addition, the molecular mechanism of response to ethanol, lignocellulosic hydrolysate inhibitors, high temperature, and osmosis stresses includes changes in the expression of regulatory proteins, such as transcription factors and sigma factors, as well as regulatory RNAs.

Some of these responses are commonly seen under several stress conditions and can be attributed to the responses that occur under multi-stress conditions, including the accumulation of Hsp proteins such as DnaK and DnaJ, which are widespread during ethanol, lignocellulosic hydrolysate inhibitors, high temperature, and osmotic stress; the accumulation of sorbitol and tryptophan during ethanol and osmotic stress situations; and the activation of the DNA repair system during high temperature and lignocellulosic hydrolysate inhibitor stress conditions. Regulatory proteins such as sigma-32 function under both high temperature and hyperosmotic stress, sigma-54 is highly expressed under both ethanol and osmotic stress, and transcription factors such as LysR, TetR, and Psp are highly expressed during furfural, ethanol, and hyperosmotic stress. These factors are attractive elements that may react with a combination of these inhibitors during ethanol fermentation.

### Impact of systems metabolic engineering on stress response and resistance in *Z. mobilis*

**Fig. 5:**
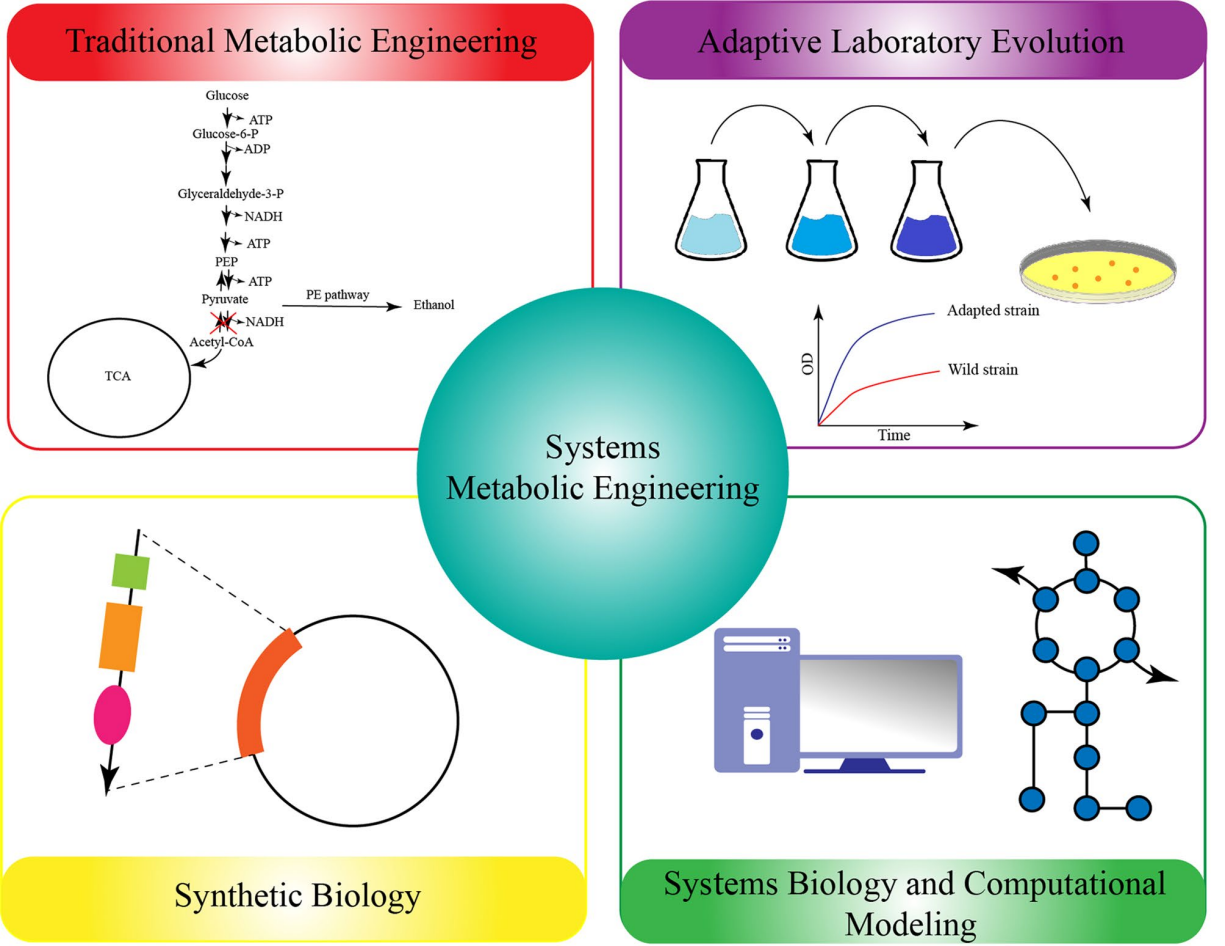
Systems metabolic engineering. Schematic view of systems metabolic engineering and its combination with classical metabolic engineering methods, systems biology and computational modeling, synthetic biology, and Adaptive laboratory evolution

As mentioned earlier, biomass refining produces several toxic and inhibitory compounds. A combination of systems biology and synthetic biology leads to the application of targeted genetic manipulation methods such as overexpression and silencing of a specific gene, DNA shuffling, gTME, and evolutionary engineering, which are seen as a promising way to improve strains and achieve significant yields and bioethanol titers [100]. In other words, current systems metabolic engineering combined with classical metabolic engineering tools, systems biology, and synthetic biology, as depicted in Fig. 5, can acquire efficient new strains with desired characteristics [18]. In addition to toxic inhibitory compounds that can impede the production of bioethanol, other notable characteristics of *Z. mobilis*, such as a narrow range of substrates, competing pathways, and the detrimental impact of carbon catabolite repression (CCR) on sugar utilization, can also influence the fermentation process [101]. It is feasible to use systems metabolic engineering techniques to create more resilient *Z. mobilis* strains for effective biofuel production. Various genetic techniques, including forward methods (NTG, transposon mutagenesis, genome shuffling, error-prone PCR) and reverse genetics (techniques guided by omics), have been employed to enhance the intrinsic inhibitor tolerance capacity of *Z. mobilis* strains [37].

We reviewed some of these methods and articles on this concept in the following sections.

#### Synthetic and systems biology

Systems biology combines biological components into a systems view and defines synthetic biology as a field in which new and artificial biological elements and pathways are constructed and designed to develop a new biological system [102]. Despite these definitions, it is worth mentioning that synthetic and systems biology are interrelated in a way that by applying knowledge of systems biology to the design of synthetic biology tools, we can in turn gain insight into systems biology [100].

One of the engineering methods that is widely used to produce recombinant strain is global transcriptional machinery engineering (gTME) that targets sigma factors and global transcription factors for strain improvement [15]. According to Lui et al., this technique designs a synthetic regulatory network with transcriptomics data which is categorized in the systems biology domain [100]. In *Z. mobilis*, gene manipulating using gTME methods elicits mutant strains with high ethanol and furfural stress tolerance. These mutations occurred in RpoD, Sigma70, a housekeeping gene controlling bacterial growth and promotor recognition, transcribed by RNA polymerase [15]. In ethanol stress, a random mutation in RpoD results in the production of strains that can tolerate 9% ethanol concentration compared to the wild types that can grow in the presence of 5% ethanol stress [15, 21].

Furthermore, glucose consumption in the mutant (0.64% of glucose remained) was more than control strain (5.43% of glucose remains) in the presence of 9% glucose in *Z. mobilis* [15]. After 24 h of incubation with 3 g/l furfural, only 22% of glucose remained in the mutant strain media, while in the control strain (non-mutant RpoD), 58% of glucose remained contact in the presence of high furfural concentration [92]. In the case of ethanol production, for ethanol and furfural mutant strains, ethanol production was about 13–14 g/l and 9.8 g/l, respectively, which was also more than the control strains [15, 92]. Mutation in sigma 70 alters ADH and PDC enzymes’ expression and activity. In the presence of 3 g/l furfural, enzyme activity changed about 3.1 to 4.6-fold compared to wild type [92].

Similarly, in the presence of 9% ethanol stress, PDC enzyme activity increased for 1.6 to 2.6 than the wild type [15]. So, it seems that the high expression of these enzymes is a kind of stress response exerted by *Z. mobilis* strains. Therefore, it can be concluded that tailoring the housekeeping sigma factor can alter gene expression and promotor recognition which results in obtaining strains with high tolerance to stress conditions.

It appears that the reprogramming of cellular metabolism by the gTME method is one of the best examples of the use of synthetic biology tools to alter microbes at the system level and generate highly tolerant strain. Another widely used technique for strain improvement is DNA shuffling. In a study by Wang et al., *Z. mobilis* strain with high tolerance to furfural and acetic acid was obtained by genome shuffling technique named *Z. mobilis* 532 (ZM532) [16]. Better growth rate, ethanol production, and glucose utilization in the presence of 7 g/l acetic acid and 3 g/l furfural were obtained, while these concentrations of inhibitors have negative impacts on wild type strains. In this study, genome shuffling revealed that several genes such as *radA* gene, which encodes DNA repair protein that controls DNA structure during stresses [103], CPSase large subunit, and arginine-tRNA ligase that controls arginine biosynthesis, increased tolerance to furfural and acetic acid stress condition [16, 103]. In addition, molecular analysis of the *Z. mobilis* 532 mutant revealed that genes and proteins involved in central carbon metabolism (Pgk, *gpmA*, glucokinase, and alcohol dehydrogenase), DNA repair system (RecF and the DNA mismatch repair enzyme) proteins, and a variety of molecular chaperones (peptidylprolyl isomerase and the Hsp20 family protein) were up-regulated, leading to tolerance in mutant strains, especially ZM532 [104]. Besides reports on overexpression of transcriptional regulators, there are also other reports on the overexpression of exogenous transcriptional factors in *Z. mobilis*. IrrE is a protein expressed by *Deinicoccus* strain in response to radiation. However, recent studies indicated that this protein can act as a regulator for recognizing the promotor region of genes such as *recA* (Recombinase A) and *pprA* (pleiotropic protein promoting DNA repair). Producing a recombinant strain that overexpresses IrrE protein can lead to tolerance to ethanol and acid and osmotic stress condition in *Z. mobilis* [105].

Another example of using synthetic biology tools to manipulate *Z. mobilis* was gene integration and transposition. Integration of *Pfu-sHSP* (under high-temperature stress conditions ) from *Pyrococcus furious* and *yfdZ, metB*, *xylA*, *xylB*, *tktA* and *talB* (amino acids biosynthesis and xylose utilization) from *E. coli* into *Z. mobilis* genome [106]. Accordingly, such a recombinant *Z. mobilis* strain can tolerate high temperatures and malnutrition. Moreover, efflux pumps seem to be another promising way to improve stress-resistant microbes [54]. In *Z. mobilis*, knocking out and downregulating of efflux pumps operon consists of ZMO0282 (efflux transporter, RND family, MFP subunit), ZMO0283 (efflux pump membrane transporter), and ZMO0285 (RND efflux system, outer membrane lipoprotein, NodT family) genes improves its tolerance toward furfural stress condition [54]. Furthermore, overexpression of *nhaA* gene that encodes hydroxylamine reductase (about 16-fold change in expression) in Acr mutant strain of *Z. mobilis* leads to overexpression of Na^+^/H^+^ antiporters and finally, improvement of strains that can tolerate and grow in the presence of 20 g/l acetic acids (an acidic-pH stress condition). In contrast, the wild type could tolerate only 12 g/l acetic acid in its growth environment [107, 108]. It can be concluded that the NhaA antiporter protein controls ion concentration and the balance of hemostasis inside and outside the cell membrane by ejecting H^+^ from the cell. Interestingly, a homolog of this protein was found in *S. cerevisiae*, and studies indicate the important effect of this protein in tolerance to acid stress [108]. Overexpression of genes can result in recombinant strains that tolerate a variety of stress levels. For instance, a recombinant *Z. mobilis* named *Z mobilis* R301, which overexpresses the *groESL* genes, can withstand multiple stress conditions, including high temperatures (40 °C), high sugar concentrations (30 g/L), and high ethanol concentrations (102.57 g/L) [109, 110]. Additionally, the overexpression of *ZMO1721*, a dioxygenase-encoding gene in *Z. mobilis* ZM4, along with NADH-dependent reductase enzymes involved in the reduction of phenolic aldehydes to phenolic alcohols, enhances the synthesis of phenolic aldehydes (4-hydroxybenzaldehyde, syringaldehyde, and vanillin), glucose consumption, and ethanol production. This system can serve as a practical synthetic biology tool to address phenolic stress conditions during bioethanol production [111]. Overexpression of some transcription factors also resulted in recombinant strains that can be active under stress conditions. In a study by Nouri et al., overexpression of transcription factors such as sigE and hfq was performed to obtain strains that can tolerate acetic acid (5 g/l) and furfural (3 g/l) under stress conditions. Comparison of the two recombinant strains revealed that recombinant strain ZM4-sigE performed better than strain ZM4-hfq in glucose utilization, ethanol production, and growth rate under furfural and acetic acid stress conditions; therefore, it appears that overexpression of regulatory factor sigE in *Z. mobilis* may be an effective way to produce a desirable strain [93]. Moreover, overexpression of *hfq* resulted in tolerance of *Z. mobilis* to ethanol stress, which was attributed to the downregulation of genes related to flagellar biosynthesis and heat stress response proteins. Additionally, there was an upregulation of genes related to sulfate assimilation and cysteine biosynthesis, leading to a reduction in reactive oxygen species induced during ethanol stress [90].

Recently, atmospheric and room temperature plasma (ARTP) has become a potent mutagenesis method that is quick, safe, and effective [112]. Using multiplex atmospheric and room temperature plasma (mARTP), mutants with enhanced resistance to acetic acid and low pH were generated. In the presence of high acetic acid concentrations, mutants AQ8-1 and AC8-9 demonstrated improved growth and ethanol production, adapting to acetic acid and low pH stressors by adjusting their NADH/NAD^+^ ratio [113].

Inhibiting competitive pathways for biofuel production, redirecting metabolic flux, and enhancing substrate utilization capability by employing CRISPR/Cas-based genome editing tools have been utilized in microorganisms to improve biofuel production [114]. Genome editing using the clustered regularly interspaced short palindromic repeats (CRISPR)-CRISPR-associated (Cas) system has been utilized to generate recombinant *Z. mobilis* strains exhibiting the desired characteristics [114, 115]. The Type I-F CRISPR/Cas system, featuring Cas3 nuclease/helicase and CRISPR–Cas12a, has been successfully detected and utilized for genome editing in *Z. mobilis* ZM4 [116, 117]. The native Type I-F CRISPR-Cas system was employed to produce the plasmid-free mutant strain of *Z. mobilis*, known as ZMNP. In comparison to the wild-type strain ZM4, *Z. mobilis* ZMNP showed improved tolerance to inhibitors, including furfural and ethanol. Additionally, several genes and proteins crucial for ROS detoxification and the oxidative stress response were found to be increased in ZMNP. These genes and proteins include glutathione reductase, MauG, thioredoxin, glutaredoxin, and alkyl hydroperoxide reductase subunit C (AhpC). It was discovered that ZMNP’s hydrolysate tolerance was influenced by increased cysteine production (including *ZMO1685* (serA1), *ZMO1684* (serC), and *ZMO0748* (cysK)) and the activation of stress response genes [118].

In silico genome-scale metabolic modeling and mathematical modeling are two of the most potent systems biology methods for creating and modifying microbes. *Z. mobilis* has been the subject of medium- and genome-scale stoichiometric reconstructions. Create microbes in such a way that the metabolic fluxes are redirected to produce more of the target products than is necessary to severely impair the behavior of the cells as a whole. Determination of intracellular fluxes based on the intricate stoichiometric connection of metabolites that make up the metabolic network is made possible by genome-scale modeling and constraint-based flux analysis. The advantage of genome-scale modeling is that it can be used in conjunction with other high-throughput methods, such as gene expression data, and provides a comprehensive prediction of the effects of genetic and environmental perturbations on cellular metabolism [119]. For instance, the genome-scale reconstructed metabolic model of *Z. mobilis* ZM4, ZmoMBEL601, comprises 579 metabolites and 601 metabolic reactions with physiological characteristics, including the ED pathway, incomplete pentose phosphate pathway, oxidative phosphorylation mechanisms, and high ethanol-producing ability [120]. In another experiment, genome-scale metabolic modeling (iZM363) of *Z. mobilis* ATCC31821 (ZM4) was performed to accurately predict the phenotypic behaviors and metabolic states in *Z. mobilis*. In addition, the functional role of the *adh* and *pdc* genes in the ethanologenic activity of *Z. mobilis* was confirmed by subsequent comparative analysis, as well as gene essentiality and flux coupling analyses [121].

A high-quality genome-scale metabolic model was used for *Z. mobilis*. The final model, called iZM516, contained data on three cell compartments, 1389 processes, 516 genes, and 1437 metabolites. Of all the reported GEMs of *Z. mobilis*, iZM516 had the highest MEMOTE score (91%), demonstrating excellent quality and accuracy was used to develop metabolic engineering techniques for biochemical production and to suggest a route for the synthesis of various biotechnological applications [122].

### Adaptive laboratory evolution

Adaptive laboratory evolution (ALE) is another metabolic engineering technique that lends itself to strain evolution and optimization through the accumulation of beneficial mutations [123]. The ALE technique was performed for phenotypic properties optimization and environmental adaptation. Applying this method in *Z. mobilis* results in introducing two mutant strains with high capability to tolerate ethanol concentrations of more than 70–90 g/l. These two mutants were designated as ER79ag and ER79ap that both have a mutation in *clpP*, *clpB*, and *spot/relA* gene; however, the position of mutations in these two strains were different [124]. ClpB is an ATP-dependent chaperone that belongs to the Hsp100-family and is a member of the AAA (ATPase associated with diverse cellular activities) chaperone. These proteins, in cooperation with other chaperones, including DnaK, DnaJ, and GrpE, are responsible for protein refolding and segregation [125]. ClpP is also a protease enzyme complex with other AAA + chaperones and denaturate misfolded or unfolded proteins [126]. It seems that mutation in these two proteins can have a crucial role in improving *Z. mobilis* tolerance to high ethanol concentration and controlling protein quality under such stress conditions. According to an investigation on mutant strains of *Z. mobilis*, it seems that *Z. mobilis* strains with mutations in *spoT/relA* perform better than other strains in a medium with 70 g/l ethanol [124]. In most bacteria, including *E. coli*, in response to a starvation condition, a stringent response system will be activated that produces and releases a compound called (p)ppGpp. A high concentration of (p)ppGpp under stress conditions results in down-regulation of genes responsible for RNA coding, flagellar synthesis, chemotaxis response, metabolite synthesis, and transporters, while up-regulated genes such as *rpoS*, *rpoH*, and *rpoE* [127]. In conclusion, the *spoT/ relA* mutation may increase the production of (p)ppGpp and accelerate the response to high ethanol concentration [124]. In another study by Yang et al., they used the ALE technique to obtain mutants of *Z. mobilis* adapted to acidic and low pH conditions. In this study, two significant mutants designated as 3.6 M and 3.5 M were derived, which grew at pH 3.8 [128].

Another mutation that leads to *Z. mobilis* tolerance to acidic pH occurred in the *ppk* gene, which encodes a phosphate kinase enzyme. The activity of this enzyme release poly P structures, which according to previous studies, control protein degradation, hemostasis, expression of sigma factor S, and DNA repair system under environmental stress condition in prokaryotes [129]. Mutation and differential expression in membrane transporters were also observable in 3.6 M mutant strain which performed better under 3.8 pH conditions. These transporters, specifically RND efflux system, ABC transporters, F1/F0 ATP synthase, are responsible for the efflux of ions, different organic compounds, and protons to reduce cytoplasm acidity and control hemostasis of the cell under acidic stress conditions [128].

Another critical target for metabolite engineering to produce *Z. mobilis* strain tolerates stress conditions performed by manipulating the *nhaA* gene [107, 108, 130]. In an experiment, a combination of two NTG and ALE techniques led to the derivation of two *Z. mobilis* mutants (ZMA-167 and ZMA-142) that were able to grow in acetic acid stress conditions (195mM acid) [130]. Genetic analysis revealed that ZMA-167 is more tolerable to acetic acid stress conditions, and this property is related to a mutation in the terminator of the *nhaA* gene. This mutation leads to overexpression of Na^+^/H^+^ antiporters and hydroxylamine reductase and finally improved resistance to acetic acid stress conditions [130]. According to other reports, the methods of ALE in *Z. mobilis* have led to the development of two mutant strains, ZMA7-2 and ZMF3-3, which can tolerate high concentrations of acetic acid (7 g/l) and furfural (3 g/l) due to overexpression of *pdc* and *adh* under such stress conditions [17]. In conclusion, it is worth saying that the ALE method can be considered a powerful tool for improving strains with desired phenotypic characteristics.

## Conclusion

*Zymomonas mobilis* is regarded as a suitable microorganism for bioethanol production through the PE pathway and activity of PDC and ADH enzymes. Although various inhibitors are produced during these metabolic pathways and fermentation processes, the physiological and molecular stress responses exerted by this species can result in significant adaptation. This series of actions can alter the cell interactome to overcome stress conditions. Changes in the molecular network of *Z. moblis* occurs at four levels of the genome, transcriptome, proteome, and metabolome during bioethanol stress. However, all four levels can directly or indirectly affect one another.

In general, under ethanol fermentation stress conditions, inhibitors such as ethanol, lignocellulosic hydrolysates inhibitors, high temperature, and osmotic stress resulted in a series of changes in gene expression and transcriptome in *Z. mobilis* and alterations in proteomes and metabolomes. Up-regulation and down-reglation of proteins including transcription factors like Lrp/AsnC family, Xre family, LysR family, LytR family, TetR family, MarR family, RpiR family, Psp family, GntR family, and LacI, as well as sigma factors such as sigma-D, sigma-N, sigma-H, sigma-F, and sigma-E are among these changes.

In addition, the expression of a variety of proteins, such as the enzymes responsible for bioethanol production, especially PDC and ADH; proteins that are responsible for controlling folded and misfolded proteins and preventing their aggregation, such as GroEL/GroES, Hsp, Lon, DnaJ, and DnaK; and proteins that activate the DNA repair system during stress conditions, such as RecA, Xse, RadA, and RadC, are influenced by these stressful environmental conditions.

Finally, alterations in the expression and repression of genes result in changes in the metabolome, specifically the contents of sugars (sorbitol), amino acids (tryptophan), and lipid membrane composition (mainly changes in lipid membrane content and structure during fermentation stress conditions that result in membrane stabilization).

Advancements in omics approaches, including genomic, transcriptomic, proteomic, metabolomics, and fluxomics, as well as computer modeling, help identify crucial components of the cell and efficient regulatory networks that facilitate stress responses. Using these data, in conjunction with the application of genetic engineering methods and the design of metabolic pathways, strains that are capable of producing ethanol and are resistant to stresses encountered during the fermentation process can be identified. Employing ALE and synthetic biology methods for *Z. mobilis* has successfully developed resistant strains. However, further investigations are required in this area. For example, future investigations could explore the application of systems metabolic engineering methods in small RNA (sRNA), the application of new genome editing tools such as CRISPR-Cas9, and the implementation of genome-scale metabolic modeling (GEM), which simulates metabolic fluxes in silico using algorithms like flux balance analysis (FBA), could be performed for developing recombinant *Z. mobilis* strains. Ultimately, comprehending the processes underlying stress response and the roles played by genes and proteins in this process aids in identifying possible targets for future metabolic engineering and systems biology research, as well as offering perspectives on the application of this bacterium in biotechnology.

## Data Availability

All data are included in the manuscript and additional information, and further queries about sharing data can be directed to the corresponding author.

## Abbreviations

GHG: Green House Gases
EMP: Embden-Meyerhof-Parnas
ED: Entner–Doudoroff pathway
GFOR: Glucose-Fructose-Oxidoreductase
HMF: Hydroxy methyl furfural
ABC: Adenine triphosphate binding cassette
RND: Resistance-Nodulation Cell-Division
MFS: Major Facilitator Superfamily
PPP: Pentose-Phosphate-Pathway
TCA: Tri-Carboxylic Acid cycle
ALD: Aldehyde dehydrogenase
ADH: Alcohol dehydrogenase
gTME: global Transcription Machinery Engineering
ALE: Adaptive Laboratory Evolution
PE: Pyruvate to Ethanol pathway
PDC: pyruvate decarboxylase
TBDT: TonB- dependent transporter
GPD: Glycerol-3-phophate dehydrogenase
TFBS: Transcription Factor Binding Site
Lrp: Leucine-responsive regulatory protein
Dam: Deoxy adenosine methylase
TFTR: TetR-Family Transcription Regulator
AAA: ATPase association with diverse cellular activities
CCR: Carbon Catabolite Repression
TAF: Transcription Associated Factor
